# Halide Engineering in Mixed Halide Perovskite-Inspired Cu_2_AgBiI_6_ for Solar Cells with Enhanced Performance

**DOI:** 10.1021/acsami.4c02406

**Published:** 2024-04-03

**Authors:** Vipinraj Sugathan, Maning Liu, Adriana Pecoraro, T. Kumar Das, Tero-Petri Ruoko, G. Krishnamurthy Grandhi, Debjit Manna, Harri Ali-Löytty, Kimmo Lahtonen, Ana Belén Muñoz-García, Michele Pavone, Paola Vivo

**Affiliations:** † Hybrid Solar Cells, Faculty of Engineering and Natural Sciences, Tampere University, P.O. Box 541, Tampere FI-33014, Finland; ‡ Department of Physics “Ettore Pancini”, University of Naples Federico II, Comp. Univ. Monte Sant’Angelo, Naples 80126, Italy; § Department of Chemical and Biological Physics, Weizmann Institute of Science, Rehovot 7610001, Israel; ∥ Smart Photonic Materials, Faculty of Engineering and Natural Sciences, Tampere University, P.O. Box 541, Tampere FI-33101, Finland; ⊥ Surface Science Group, Photonics Laboratory, Tampere University, P.O. Box 692, Tampere FI-33014, Finland; # Faculty of Engineering and Natural Sciences, Tampere University, P.O. Box 692, Tampere FI-33014, Finland; ∇ Department of Chemical Sciences, University of Naples Federico II, Comp. Univ. Monte Sant’Angelo, Naples 80126, Italy

**Keywords:** perovskite-inspired materials, halide engineering, Cu_2_AgBiI_6_, traps, solar cells, efficiency

## Abstract

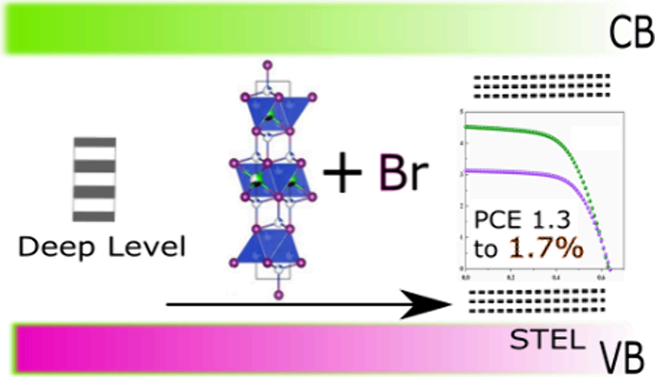

Cu_2_AgBiI_6_ (CABI) is a promising perovskite-inspired absorber for solar cells due to its direct band gap and high absorption coefficient. However, the nonradiative recombination caused by the high extrinsic trap density limits the performance of CABI-based solar cells. In this work, we employ halide engineering by doping bromide anions (Br^–^) in CABI thin films, in turn significantly improving the power conversion efficiency (PCE). By introducing Br^–^ in the synthetic route of CABI thin films, we identify the optimum composition as CABI-10Br (with 10% Br at the halide site). The tailored composition appears to reduce the deep trap density as shown by time-resolved photoluminescence and transient absorption spectroscopy characterizations. This leads to a dramatic increase in the lifetime of charge carriers, which therefore improves both the external quantum efficiency and the integrated short-circuit current. The photovoltaic performance shows a significant boost since the PCE under standard 1 sun illumination increases from 1.32 to 1.69% (∼30% relative enhancement). Systematic theoretical and experimental characterizations were employed to investigate the effect of Br^–^ incorporation on the optoelectronic properties of CABI. Our results highlight the importance of mitigating trap states in lead-free perovskite-inspired materials and that Br^–^ incorporation at the halide site is an effective strategy for improving the device performance.

## Introduction

Owing to their outstanding optoelectronic properties and low-cost solution processing,^1−3^ lead halide perovskites have been recently explored as the most promising light absorbers for next-generation solar cells. However, using lead halide perovskites raises high toxicity concerns, which blocks their path to commercialization.^4,5^ Hence, there is a need to develop more efficient, lead-free alternatives that match the optoelectronic capabilities of lead halide perovskites.

To this end, earth-abundant and less toxic perovskite-inspired materials, viz., lead-free bismuth-based halides, have attracted rising attention due to their favorable optoelectronic properties and high structural stability.^6,7^ One type of this material is the Cu–Ag–Bi–I quaternary pnictogen-based halide, namely, Cu_2_AgBiI_6_ (CABI), which has gained remarkable research attention in recent years. CABI has been explored for solar cells and other optoelectronic applications due to its very high absorption coefficient (>1 × 10^5^ cm^–1^) and direct band gap nature.^8−14^

However, the performance of CABI-based solar cells is still far from reaching its full potential, though considerable attempts have been made in this direction.^15^ The major performance constraint is ascribed to the low-quality morphology of the CABI films, while the so-called point defects significantly contribute to the formation of undesired charge carrier traps and, hence, big losses in photocurrent.^13,15^ Our recent work has addressed some of these issues, implementing Sb–Bi alloying in CABI to effectively suppress the nonradiative recombination, in turn leading to a considerable improvement in the photovoltaic performance.^13^ The defects in CABI are formed due to the I-poor environment.^9^ A similar situation has been observed as a source of interstitial deep-level point defects (DLD), leading to nonradiative recombination in other materials such as FAPbI_3_.^16,17^ These defects are mainly generated within the band gap when the formation energy of the defects is high.^18^ On the other hand, there is a possibility to form a shallow transition energy level (STEL) when the point defect has low formation energy. STEL is closely related to the conduction band minimum (CBM) or valence band maximum (VBM), thus enabling charge carrier trapping.^18^ The STEL is often responsible for the defect tolerance of conventional perovskite materials.^19^ The shallow-level defects arising from STEL can be employed for charge carrier extraction upon rational defect engineering, which can be achieved by using controlled doping strategies.^20^ An effective approach to improve the film morphology while inhibiting the formation of defects in lead halide perovskite solar cells is the partial or complete replacement of iodine by bromine.^21−24^ Br^–^ anions have been employed as effective dopants at the halide site to improve the defect tolerance of the perovskites. Meggiolaro et al. predicted that Br^–^ can substitute I^–^, resulting in a shift at the trap energy levels toward the VBM. This reveals that the interstitial DLD can be effectively converted into shallow-level defects for enhanced photocurrent.^19^

In this work, we propose a strategy that can possibly suppress the defects associated with the halide (I) site in the lattice of CABI, by partially replacing I^–^ with Br^–^ in its composition. Halide engineering is conducted by synthesizing a series of CABI compositions through the partial replacement of iodide with bromide at the halide site. The effect of mixed halides on their corresponding optoelectronic properties is systematically investigated. The surface coverage is fine-tuned, with a significant reduction in the grain boundaries. More interestingly, an obvious reduction in the number of self-trapped states of charge carriers is observed, which is attributed to the decrease in the amount of DLD, instead of formation of shallow defects. The use of CABI-*x*Br (where *x* denotes the percentage of Br^–^ at halide sites) as the light absorber effectively increases the device performance with the highest power conversion efficiency (PCE) of 1.69%, an almost 30% relative enhancement compared to that of reference devices based on pristine CABI (1.32%). Our theoretical and experimental results highlight the significance of defect engineering based on optimally mixed halides for enhancing the performance of perovskite-inspired solar cells.

## Results and Discussion

The reference sample, pristine Cu_2_AgBiI_6_, is referred to as “CABI ref” hereafter. Varying the amounts of Br^–^ at I^–^ sites leads to the targeted mixed halide compositions, which are referred to as CABI-5Br, CABI-10Br, CABI-20Br, and CABI-40Br, respectively, for 5, 10, 20, and 40% of Br^–^ mixing with I^–^. The different percentages presented here are defined based on the nominal ratios during the precursor preparation, as described in the Experimental Procedures.

Here, CABI-5Br represents the smallest amount of Br^–^ that we investigated, CABI-10Br represents the optimum composition that yields the best results in solar cells, and CABI-40Br represents the composition with the highest amount of Br^–^. A further increase (above 40%) in the amount of Br^–^ leads to the formation of impurity phases; thus, our experiments did not focus on higher amounts of Br^–^. Though several other intermediate compositions were tested, the initial experiments revealed more prominent similarities in characteristics than the reported ones; thus, detailed experiments for those compositions are not included in this work.

**Figure 1:**
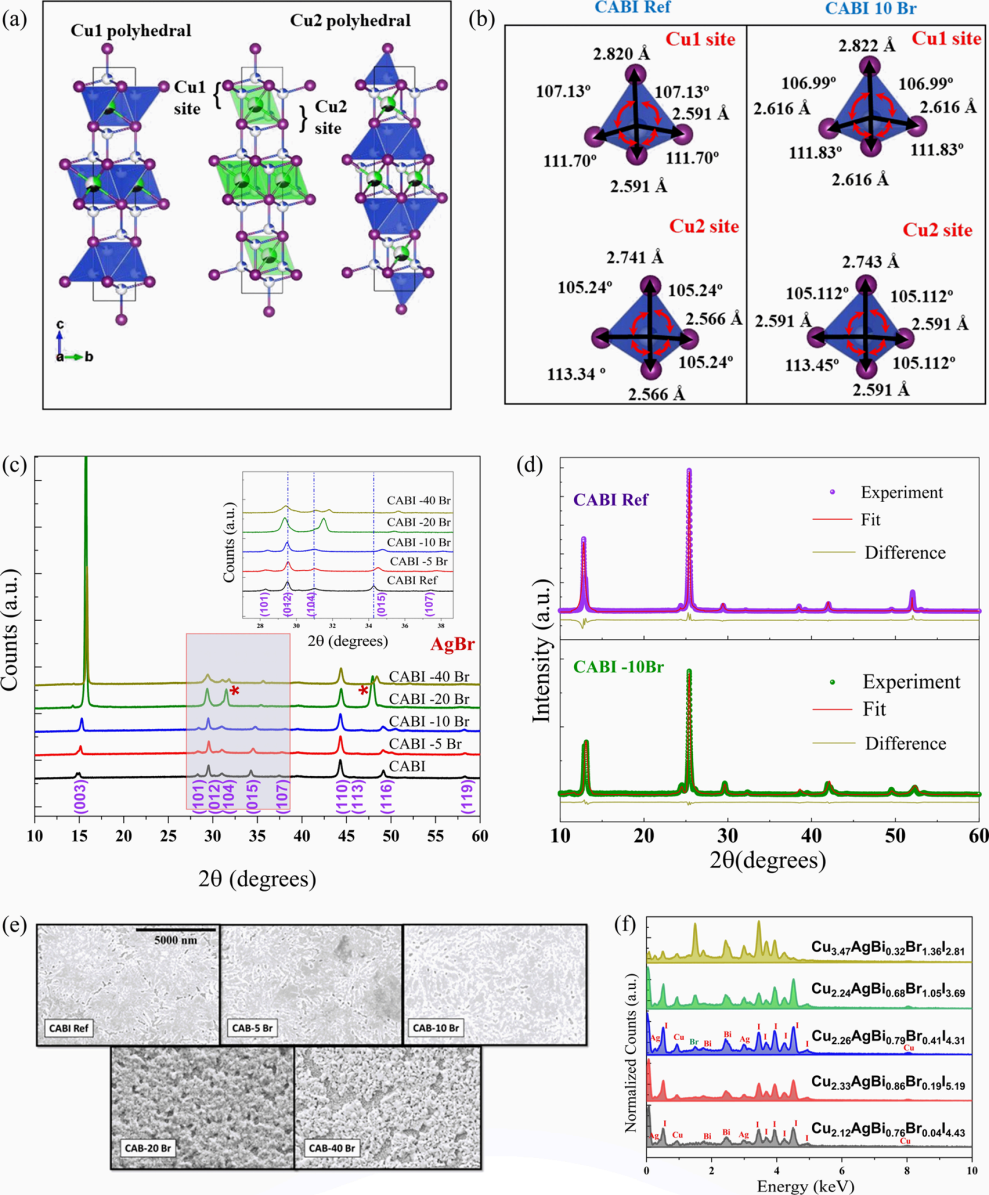
(a) Crystal structure of CABI, (b) comparative bond metrics for CABI and CABI-10Br, (c) comparative XRD plots for varying amounts of Br^–^, (d) XRD refinement results for CABI and CABI-10Br, (e) SEM images illustrating the film morphologies for the different compositions, and (f) comparative EDX plot for the examined compositions.

CABI contains an iodide sublattice to achieve a cubic closely packed structure,^9^ as presented in Figure 1a. The Ag^+^ and Bi^3+^ cations contribute to the octahedra by forming a distorted CdCl_2_-like arrangement. The overall structure resembles a layered 2D arrangement with alternating layers separated by octahedral vacancies. Figure 1b depicts the difference in the estimated bond metrics of the two Cu sites. The addition of Br^–^ leads to a reduction in distortion angles, while there is a slight elongation of the bonds. This change in the structural parameters was further investigated using X-ray diffraction (XRD) analysis.

XRD measurements (Figure 1c) reveal that there is a linear shift in peaks with an increased amount of Br^–^ to higher 2θ values, indicating lattice contraction due to partial replacement of iodide with bromide. The lattice spacing steadily reduces as I atoms are gradually replaced with smaller Br atoms, as evident by the shift of the diffraction peaks corresponding to the (015) and (104) planes.^25^ The supercell volume decreases due to the substitution of longer I–Bi bonds by shorter Br–Bi bonds. The theoretical investigation of the structural and electronic features of Br-doped CABI was performed by using density functional theory (DFT) calculations. Starting from the parent CABI structure with Cu_2_AgBiI_6_ composition, modeling was done for Br doping by partially replacing I atoms with Br to obtain different bromine contents close to the experimental ones, i.e., 5 (CABI-5Br), 10 (CABI-10Br), 20 (CABI-20Br), and 40% (CABI-40Br).

Detailed structural models are presented in Figure S1 in the Supporting Information (SI). Lattice constants obtained for the PBE-TS relaxed structures are summarized in Table 1. As the amount of bromide is increased, the lattice dimension decreases linearly (from *a* = *b* = 4.35 Å, *c* = 20.93 Å for CABI ref to *a* = *b* = 4.27 Å, *c* = 20.35 Å for CABI-40Br). This confirms the decrease of the cell volume with an increasing Br content, as observed in the XRD patterns. It can also be observed here that with higher amounts of Br^–^(>10%), impurity peaks at 31.5 and 47.5° start to appear, and they correspond to the (200) and (220) planes, respectively, of AgBr. To validate the structural changes, Rietveld refinement was performed using X’Pert HighScore plus software on the high-resolution XRD patterns of CABI and CABI-10Br (as a representative of compositions with a low concentration of Br) samples using a standard reference file (CCDC2013668).^9^Figure 1d portrays the comparative refinement results for the two compositions, revealing that for both films, no trace of secondary phase is observed. The lattice parameters of the CABI are determined to be *a* = *b* = 4.33 (Å) and *c* = 21.19 (Å), which decrease to *a* = *b* = 4.29 (Å) and *c* = 20.95 (Å) for CABI-10Br, respectively. This suggests that the Br^–^ ion indeed replaces the I^–^ ion in the crystal structure. Calculated *a* (*a* = *b*) and *c* lattice constants for pristine CABI exhibit 0.5 and 1.2% deviations from the corresponding Rietveld refined values (reported in parentheses in Table 1), while 0.9 and 0.2% deviations are observed for CABI-10Br with respect to the corresponding experimental values. The distortion parameters were estimated for the two compositions (Table S1), showing that with the addition of Br, the mean metal (Ag and Bi) halide (I and Br) distances are shorter than those of pure CABI, as also observed experimentally. The other distortion parameters are smaller for models with bromine, indicating more regular structures upon Br incorporation, which agrees with the experimental observation. The change in structural properties makes a significant effect on the material’s optical and electrical properties.^26^ A phase transition can induce this shift to be considerably larger, which can be seen in this case with higher amounts of Br (>10%).^27^ Hence, it can be concluded that for lower concentrations of Br (<10%), the phase is retained. Figure 1e depicts the variation in the film morphology with the increase in Br concentration. As the amount of Br in the precursor increases, the crystals start to agglomerate. Analysis of the surface morphology of CABI and CABI-10Br films (Figure S2) reveals that, with addition of Br, a fusion phenomenon among neighboring crystal groups occurs, which can inhibit the island formation effect commonly observed for pristine CABI films. With 10% Br, the fusion among the crystals leads to enhanced surface coverage and a reduced number of pinholes compared to the case of pristine CABI. Energy-dispersive spectroscopy (EDX) analysis was conducted to confirm the bulk compositions, shown in Figure 1f as a comparative plot. The calculated compositions and Br content in the sample corresponding to each composition are summarized in Table S2. All of the different compositions have an excess of Cu and Ag due to the loss in BiI_3_ during the film formation. This loss in BiI_3_ has also been observed in previous experimental procedures that involve BiI_3_ in the synthesis of pristine CABI.^9,28^ With a small amount (≤10%) of Br^–^ introduction, the overall composition of CABI remains the same except that the amount of I^–^ decreases. This confirms the successful replacement of I^–^ by Br^–^ ions in the bulk. For larger amounts (≥20%) of Br^–^, the composition is disturbed, indicating the formation of impurity phases as also confirmed by our XRD analysis. The actual composition for CABI was estimated to be Cu_2.12_AgBi_0.76_Br_0.04_I_4.43_, while that of CABI-10Br was Cu_2.26_AgBi_0.79_Br_0.41_I_4.31_. Br accounts for 8.7% of the halide content in CABI-10Br. The EDX mapping data shown in Figure S3 demonstrate a uniform distribution of all elements, which further confirms the formation of phase-pure films with no observed segregation of impurities in the bulk. The surface composition of CABI and CABI-10Br was analyzed by X-ray photoelectron spectroscopy (XPS), indicating that the Br percentage of the halide was ∼8.5%, which is in good agreement with the bulk composition. The XPS survey plot and high-resolution spectra for the different elements are presented in Figure S4. Table S3 summarizes the surface composition for different elements as obtained from XPS analysis in CABI ref and CABI-10Br films.

**Table 1:** Lattice Parameters of the Rhombohedral CABI-Br Unit Cell with Different Br Contents^a^

lattice parameters (Å)	CABI ref	CABI-5Br	CABI-10Br	CABI-20Br	CABI-40Br
*a* = *b*	4.35 (4.33)	4.34	4.33 (4.29)	4.30	4.27
*c*	20.93 (21.19)	20.76	20.91 (20.95)	20.69	20.35

a All structures are calculated at the PBE-TS level of theory. Values in parentheses are experimental Rietveld refined values.

The optoelectronic properties of the different compositions were examined to understand the effect of addition of Br^–^ in CABI. A recent report regarding the electronic structure of CABI shows that the CBM is dominated by Bi 6p and I 5p states, similar to AgBiI_4_ and BiI_3_.^29^ In contrast, the VBM is dominated by Cu 3d states, mixed with the I 5p states. Optical transitions near the band gap edge of Cu_2_AgBiI_6_ involve considerable Cu 3d to Bi 6p/I 5p character. Br introduction can lead to Br 5p states at the valence band edge being slightly lower than the I 5p energy states.^25^

**Figure 2:**
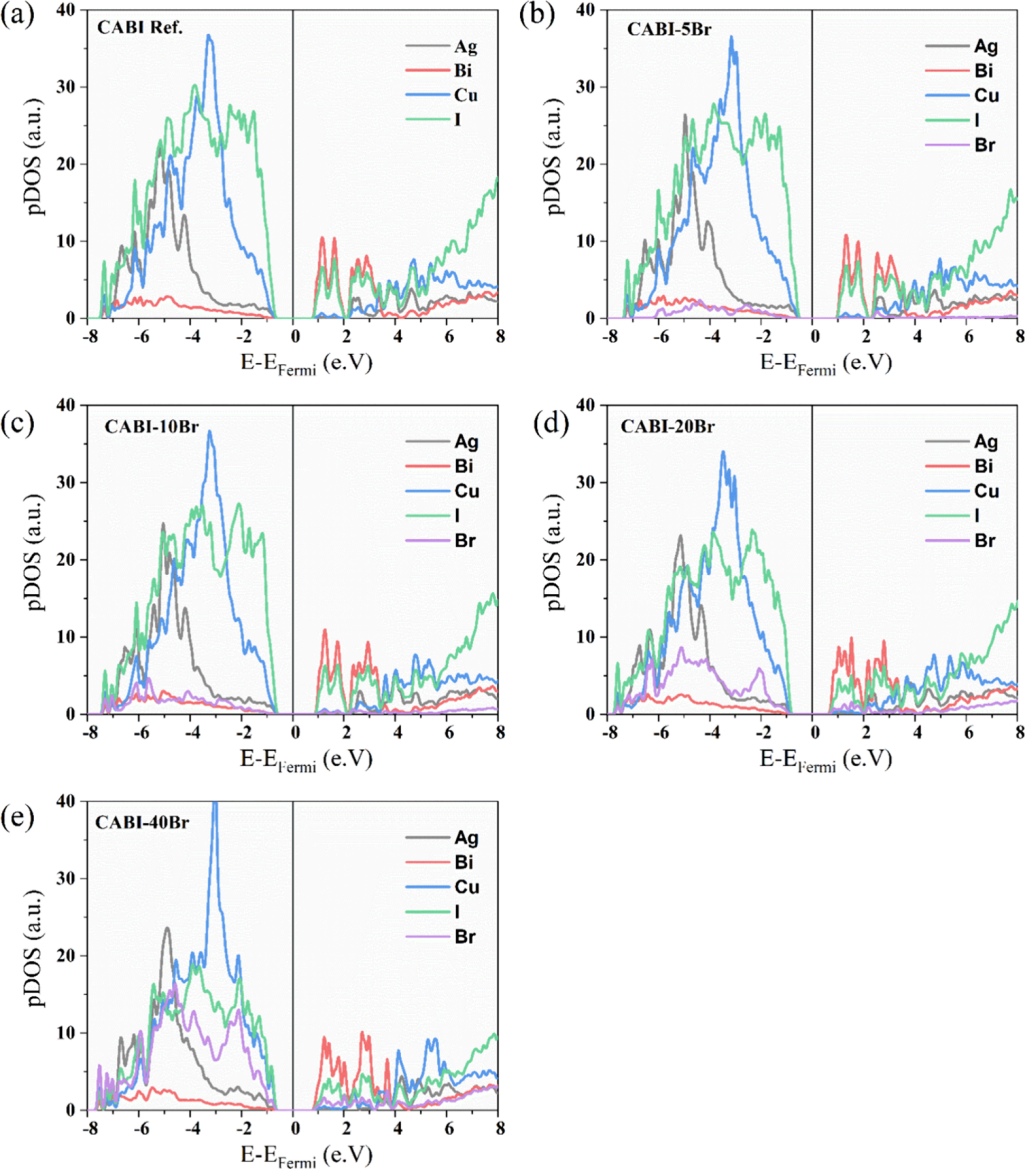
Atom-projected density of states (pDOS) calculated at the PBE0 level of theory for the CABI-Br cell models for (a) CABI ref, (b) CABI-5Br, (c) CABI-10Br, (d) CABI-20Br, and (e) CABI-40Br.

**Figure 3:**
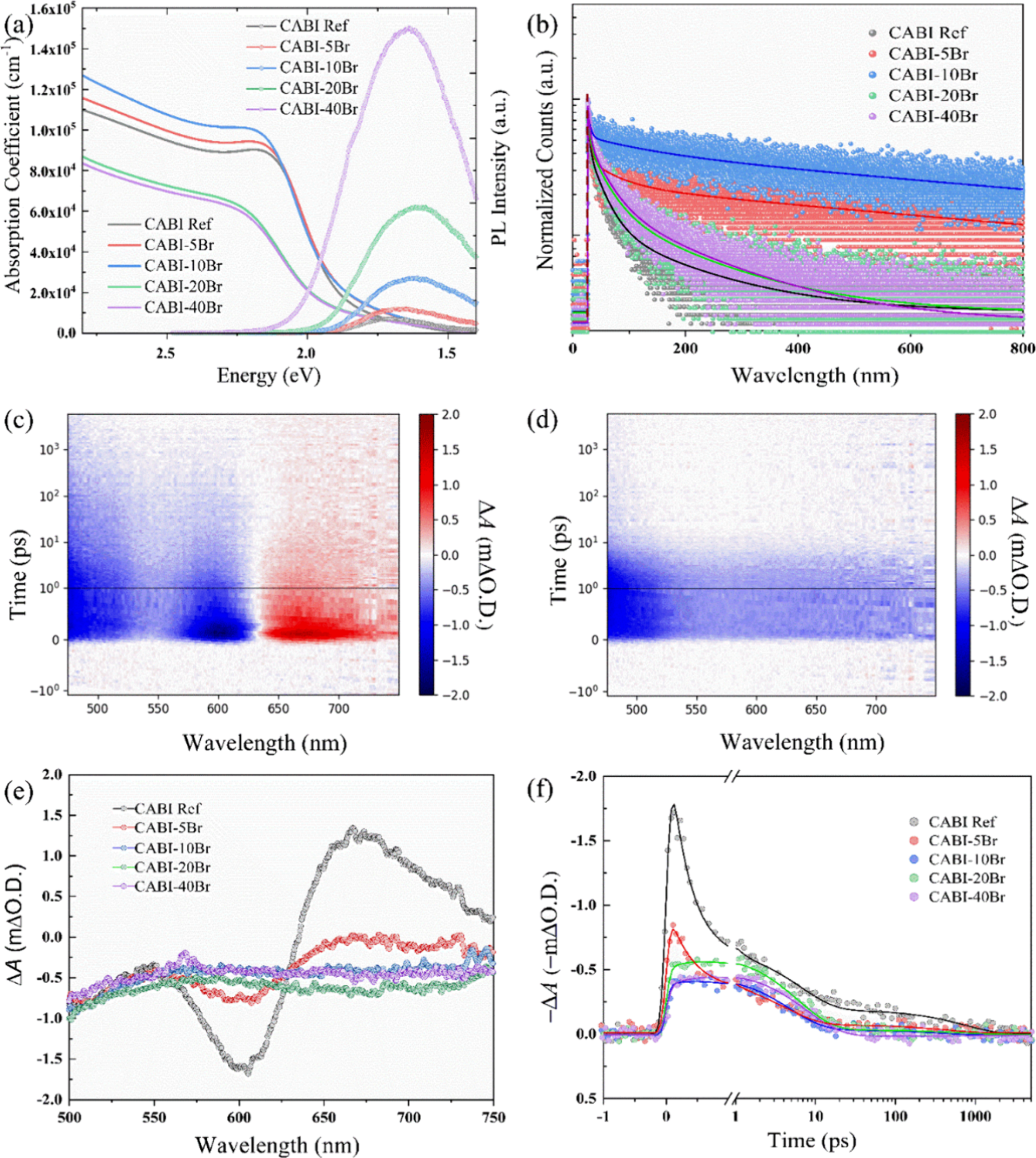
(a) UV–visible spectroscopy plot and steady-state photoluminescence (ss-PL) spectra, monitored at 680 nm. (b) Time-resolved PL (TRPL) decays of pristine CABI and CABI-*x*Br (*x* = 5, 10, 20, and 40) films, excited at 405 nm. Transient absorption (TA) mapping images of (c) pristine CABI and (d) CABI-10Br films, excited at 400 nm with an excitation energy density of 20 μJ cm^–2^. (e) TA spectra at 0.2 ps after excitation and (f) inverted photobleaching (PB) decays (monitored at 600 nm) of pristine CABI and CABI-*x*Br. Solid lines in (f) represent the fits corresponding to a triexponential function: ΔO.D. = *A*_0_ + *A*_1_ exp(−*t*/*t*_1_) + *A*_2_ exp(−*t*/*t*_2_) + *A*_3_ exp(−*t*/*t*_3_). ΔO.D. is the change in the optical density.

Electronic feature evaluation via the projected density of states (pDOS) is shown in Figure 2. The main contribution to the valence band (VB) of CABI comes from copper and iodine, while the conduction band (CB) is dominated by bismuth and iodine p states, in agreement with a previous report.^9^ Our theoretical results suggest that, with the addition of bromine, Br states populate the VB. The experimentally obtained UV–visible absorption spectra (Figure 3a) reveal that pristine Cu_2_AgBiI_6_ has an absorption coefficient of around 1 × 10^5^ cm^–1^, which is in good agreement with the reported values.^9^ No obvious changes upon the addition of Br are observed in the absorption spectra. However, the absorption coefficient initially increases with 5 and 10% Br^–^ incorporation and then begins to decrease, leading to CABI-10Br having the largest absorption coefficient among the compositions.

We attribute this to the improvement in the surface morphology of the films. The Tauc plots presented in Figure S5a in the SI correspond to a direct allowed transition with a band gap of 1.95 eV for CABI. However, the indirect band gap of CABI is determined to be smaller (1.88 eV) than the direct band gap (Figure S5b). A clear distinction between the band gaps corresponding to direct and indirect transitions is not straightforward, likely due to the morphology of films. Scattering from the rough film surface can lead to prolonged tails in the absorption spectra, decreasing the determined indirect band gap value.^30^ The smaller indirect band gap can also result from point defects in the material.^31^ The band gaps recorded for the different compositions are presented in Table S4. With increased amounts of Br, the direct band gap increases only marginally. The band gap determined for an indirect transition also increases, possibly indicating an improvement in the film morphology by lowering the number of point defects. Theoretical band gaps calculated at the PBE0 level of theory are collected in Table S5. The theoretical values are slightly smaller than the experimental ones and can reproduce the direct/indirect experimental characteristics only for CABI-10Br. The computational results predict, in agreement with experiments, an increase in the band gap values with addition of Br. The morphology, however, does not seem to improve much beyond 10% Br compositions. We speculate that the broadening in indirect band gaps can be ascribed to the reduction in point defects that causes the absorption tail to diminish. However, since Tauc analysis ignores the exciton contribution close to the band edge, an Elliott model was used to fit the experimental absorbance spectrum and characterize the electronic structure of the films.^32^ The transmittance and diffused reflectance spectra were collected, and the absorption spectra were derived using the expression *A* = −log[*T*/(1 – *R*)].^33^Figure S6a–e shows the fit graphs with the continuum and excitonic components included for the films with different amounts of Br. The band gap (*E*_g_) estimated for all the films is 2.2 eV, consistent with a previous report.^14^ It is observed that the exciton binding energies decrease with an increased amount of Br (Figure S6f). This decrease in exciton biding energy facilitates charge carrier generation and hence can contribute to improved photocurrents in solar cells.^14^ A decrease in exciton binding energy also indicates larger carrier lifetimes and reduced recombination losses.^34^ The blueshift in the excitonic peaks explains the change in the band gap observed from the Tauc plot analysis.

To gain additional insight into the optical properties, we measured the steady-state photoluminescence (ss-PL) spectra and time-resolved PL (TRPL) decays for the pristine CABI and CABI-*x*Br films. Figure 3a shows the room-temperature PL spectra of the different CABI films that were excited at 405 nm. Notably, the PL intensity increases with an increase in the amount of Br^–^. This may suggest that the PL intensity associated with STEL can be enhanced in the presence of deep-level trap centers.^35^ A longer radiative lifetime can indicate a curing of DLD due to reduced nonradiative recombination.^36^ Further, the steady-state photoluminescence (ss-PL) spectra for the pure CABI and CABI-10Br films were analyzed in detail to extract more fundamental information. A broad asymmetric emission band is observed for all PL spectra, indicating that a multiphasic electron transition is involved in the whole process of charge carrier relaxation. Notably, the PL peak exhibits a redshift (see Figure 3a) from ∼1.72 (pure CABI) to ∼1.63 eV (CABI-xBr) when a higher amount of bromine is blended, e.g., *x* = 20 and 40, accompanied by a dramatic increase in the PL intensity. This suggests that the Stokes shift is even enlarged along with the increase in the mixed amount of Br by considering the blueshifted exciton peak in the absorption spectra (Figure 3a). To further clarify the multiple components for the asymmetric emission bands, we deconvoluted each PL spectrum into three Gaussian bands (Figure S7a,b), centered around 1.75 (P1), 1.60 (P2), and 1.5 eV (P3). The three emission peaks (P1–P3) can be assigned to band-to-band emission (P1), which has been observed for CABI materials^15^ or similar PIMs,^37^ shallow-level emissions (P2), being close to the band-to-band emissions,^38^ and deep-level emission (P3).^39^ It is observed that the deep-level emission (P3) is quite evident in the PL spectrum of pure CABI, while it diminishes for the CABI-10Br sample. This can be attributed to reduction in deep traps with addition of Br^–^ in CABI. The contributions from the shallow-level emissions (P1) and band-to-band emission (P3) together play a major role in contributing to the PL spectra once the bromine is introduced into the iodine site. While the contribution from shallow-level emission remains more or less constant here, the contribution from band-to-band emission is boosted for the CABI-10Br samples. Since the shallow trap level is the result of transition energy levels close to the valence band maximum (VBM) or conduction band minimum (CBM), the carriers trapped in them can easily diffuse back into the VBM or CBM.^40,41^ This in turn, contributes to band-to-band emissions. To further ascertain this phenomenon, we carried out excitation-dependent PL measurement, as a separate state that emits at a different energy is excited by a new excitation wavelength when the excitation wavelength is altered and there is a dispersion of distinct emissive states.^38^ The results are plotted as shown in Figure S7c,d.

We investigated the excited-state dynamics of these samples by measuring time-resolved PL (TRPL) decays (Figure 3b). All TRPL decays were fitted with a 3-exponential function, and the fitting results are summarized in Table 2. To compare the TRPL decays, we estimated the average lifetime for each sample. The PL decay of CABI-10Br exhibits the longest lifetime (*t*_avg_ = 567.5 ns), while CABI ref (*t*_avg_ = 20.4 ns) and CABI-40Br (*t*_avg_ = 27.3 ns) samples have the fastest radiative lifetimes. The changes in lifetime suggest that small amounts of mixed Br (i.e., 5–10%) at the I site result in the longest radiative lifetimes due to a reduction in trap states responsible for nonradiative recombination, whereas incorporating more Br (e.g., 20 or 40%) increases nonradiative recombination due to the formation of multiple phases in the material. We show that CABI-10Br is the most favorable composition for suppressing DLD, resulting in efficient charge transfer in devices. We speculate two possible reasons to explain this change: (i) The introduction of Br at the I site significantly increases the formation energy of self-trapped excitons, leading to the decreased number of self-trapped excitonic states, which is related to the reduced lattice distortion upon mixing of Br with I (see previous XRD and DFT analysis). (ii) The involvement of Br can effectively fill the iodide vacancies, sufficiently reducing the number of DLD or traps, thus hindering the nonradiative recombination.

**Table 2:** Fitting Results of the TRPL Data

	%*A*_1_	*t*_1_ (ns)	%*A*_2_	*t*_2_ (ns)	%*A*_3_	*t*_3_ (ns)	a*t*_avg_ (ns)
CABI ref	63.5	6.1	31.3	52.1	5.1	486.3	20.4
CABI-5Br	54.7	6.3	16.3	62.0	29.0	1081.0	301.9
CABI-10Br	34.7	5.3	15.7	121.2	49.7	1183.0	567.6
CABI-20Br	55.6	9.6	36.3	72.6	8.1	482.7	31.8
CABI-40Br	59.9	11.9	34.0	82.1	6.2	504.7	27.2

a .

To further assess the photophysical properties of CABI-Br films with different amounts of Br, we conducted ultrafast transient absorption (TA) by exciting the samples at 400 nm with an excitation energy density of 20 μJ cm^–2^. Figure 3c,d compares the 2D TA mapping images of pristine CABI and CABI-10Br films, while the TA spectra at 0.2 ps for all compositions are shown in Figure 3e. The 2D TA mapping images of CABI-5Br, CABI-20Br, and CABI-40Br films are shown in Figure S8 for comparison. A positive excited-state absorption (ESA) band with a fast lifetime of 0.31 ps located at 630–750 nm in pristine CABI decreases in amplitude for CABI-5Br and is not observed for any sample with a larger Br content. Based on previous TA studies of CABI films,^11,15,42^ this ESA band is assigned to be the existence of STEL states, suggesting that the introduction of Br at the I site can indeed mitigate or even eliminate the formation of self-trapped excitons at the lattice level. In addition, a photobleaching (PB) band is observed centered at 600 nm for pristine CABI and, to a lesser extent, CABI-5Br. Samples with a higher Br^–^ content illustrate only weaker broad and featureless PB over the whole monitoring range. The PB band in CABI and CABI-5Br does not match with the steady-state absorption onset but instead overlaps with the excitonic peak position visible as a shoulder in the absorption spectra in Figure 3a. This reveals that the absorption spectrum of CABI or CABI-Br films consists of a continuum of states and an excitonic contribution, which are not well-resolved in the steady-state absorption spectra.^9^ Therefore, the localized PB band is attributed to the change of the excitonic contribution stemming from the relaxation of charge carriers to the band edge (either direct or indirect) upon the photoexcitation in CABI and CABI-5Br.^43^ The PB decays at 600 nm are shown in Figure 3f along the 3-exponential fits (see the summarized fitting results in Table S7 in the Supporting Information). The excitonic PB has a fast decay component with a lifetime of 0.31 ps, matching the lifetime of the positive ESA band. On the other hand, the samples with a larger Br^–^ content decay biexponentially and do not contain a subpicosecond lifetime decay component that we assign to the excitonic feature. Furthermore, the PB decay of CABI-10Br shows the longest average lifetime (*t*_avg_ = 155 ps), while that of CABI-40Br exhibits the shortest average lifetime (*t*_avg_ = 79.09 ps). This is consistent with the trend of TRPL decay lifetimes, confirming that the efficient hindering of nonradiative recombination can be achieved when mixing 10% Br at the I site, further asserting suppression on DLD.

We performed further theoretical validation by using the CABI-10Br model as a representative framework to investigate the impact of halide vacancies on the electronic properties of these materials. We model the point defects by extracting a single neutral I/Br atom from the cell with a focus on three distinct local environments. Specifically, our analysis encompasses the scenario of a Br vacancy, along with two types of iodine vacancies, dependent on the local octahedra composition—full iodine content (VI(I)) and mixed I/Br composition (VI(Br)). The three considered models are depicted in Figure S9, together with the starting pristine model for comparison. For each model, we have quantified the ease to form the defect through calculations of the energy of vacancy formation, evaluated as
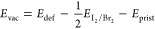
1where *E*_def_, *E*_I_2_/Br_2__, and *E*_prist_ are the total energies of the defective system, the halogen molecule in the gas phase, and the pristine structure, respectively. Values obtained at the PBE0 level of theory for the three models are collected in Table 3.

**Table 3:** Vacancy Formation Energies Calculated at the PBE0 Level of Theory, According to Equation 1

	*V*_Br_	*V*_I(I)_	*V*_I(Br)_
*E*_vac_ (eV)	2.68	2.18	2.10

Our results predict iodine vacancies to be easier to form than bromine, as expected from the strength of Bi–I and Bi–Br bonds.^44^ This observation further asserts that the probability of formation of vacancies can be suppressed by addition of Br^–^, which in turn leads to suppression of DLD. Also, for the mixed I/Br composition, the formation energy of iodine vacancy decreases, which can be assigned as a reason for the formation of STEL.^18^ For each structure, we have also analyzed the effects of the defect on the electronic structure via projected density of state (pDOS) calculation, as depicted in Figure S10. It is observed that the iodine vacancy in mixed I/Br octahedra results in new states close to the conduction band, while this is not observed in the case of iodine vacancies in CABI. The first-principles calculations and analysis further validate the suppression of DLD and formation of STEL in CABI with addition of Br^–^.

To verify the effect of Br incorporation on the photovoltaic performance, the CABI-*x*Br-based solar cells were fabricated with the structure “FTO/c-TiO_2_/mp-TiO_2_/CABI-*x*Br/Spiro-OMeTAD/Au”. The absorbers CABI ref and CABI-10Br were chosen for comparative analysis. Devices were also fabricated for all the other compositions, and from the device statistical analysis, CABI-10Br shows the best performance. The PCEs for all cases are compared in Figure S11, depicting a statistical distribution plot for 10 devices of each type. The poor performance of the solar cells with CABI-20Br and CABI-40Br absorbers can be attributed to the presence of AgBr impurities.

**Figure 4:**
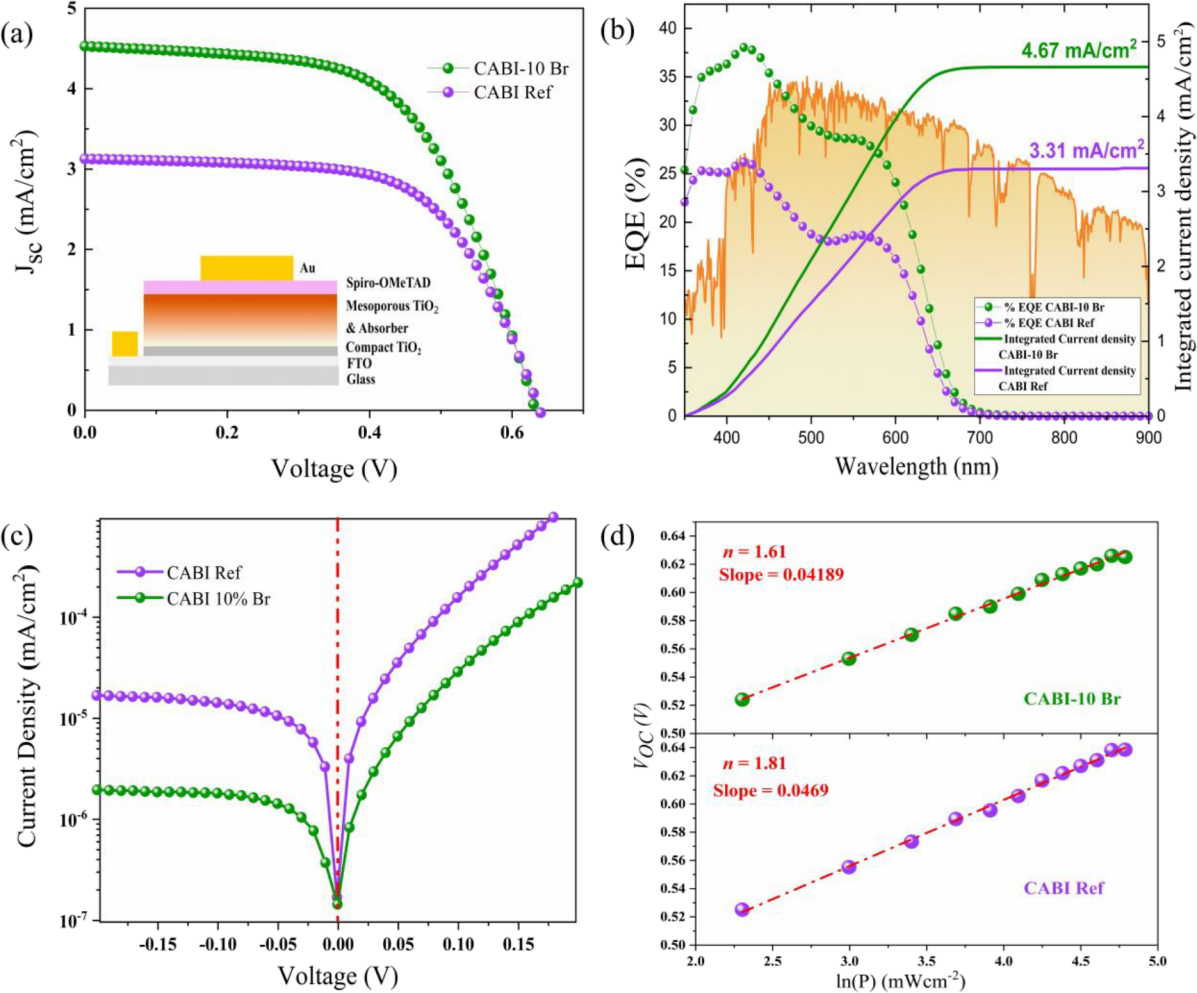
(a) *J*–*V* plots under standard 1 sun illumination, reverse scan from 0.7 to 0 V, (b) EQE spectra and integrated current density illustrated with 1 sun spectra, (c) dark *J*–*V* plots, and (d) light intensity-dependent *V*_OC_ for CABI- and CABI-10Br-based devices.

The fabrication details are described in the Experimental Procedures. The architecture and methods were chosen based on previously optimized procedures for CABI solar cells.^8,10^Figure 4a depicts the current density (*J*)**–**voltage (*V*) curves (reverse bias scans) for CABI- and CABI-10Br-based devices measured under 1 sun illumination (AM 1.5G, 100 mW cm^–2^). The statistical distribution of the device parameters obtained for 15 devices is shown in Figure S12. Table 4 presents the device parameters for CABI- and CABI-10Br-based solar cells under 1 sun illumination. The champion CABI ref device shows a PCE of 1.32%, while the CABI-10Br one reaches the highest PCE of 1.69%. This is an improvement over most of the previously reported CABI-based devices.^8−10,15^ Based on statistical analysis, there is an improvement in the fill factors (from 61.7 to 65.9%) for the devices with Br incorporation compared to the reference case.

**Table 4:** Photovoltaic parameters for CABI- and CABI-10Br-based solar cells under 1 Sun of illumination

	*J*_sc_ (mA cm^–2^)	*V*_oc_ (V)	FF (%)	PCE (%)
CABI ref	3.05 ± 0.32	0.65 ± 0.019	61.78 ± 2.35	1.22 ± 0.054
CABI-10Br	4.10 ± 0.28	0.64 ± 0.011	65.86 ± 1.38	1.58 ± 0.055

Also, the *J*_sc_ is significantly higher for the CABI-10Br-based devices (4.52 mA cm^–2^) compared with that of the CABI ref (3.13 mA cm^–2^), attributed to improved light harvesting and the reduced nonradiative recombination with less trapped and/or self-trapped states, which have been observed in previous TA measurements.^15^ The presence of STEL close to the VB populates the VB contributing to an increase in the overall *J*_sc_.

The presence of shallow defects can contribute to voltage losses and hysteresis in solar cells.^45^ The presence of both deep and shallow defects can deteriorate the solar cell performances, particularly the *V*_oc_.^46^ However, when applied in solar cells, the hysteresis for the CABI-10Br-based devices remained unchanged when compared to that of the CABI devices. Figure S13 depicts the *J–V* curves obtained for the forward bias scans of CABI- and CABI-10Br-based devices. The corresponding PCEs were 1.28 and 1.64%, respectively, which account for hysteresis indices of 0.969 and 0.970 for CABI- and CABI-10Br-based devices, respectively. Though the addition of bromine leads to negligible voltage losses, it can be considered as a trade-off for the significant gain in current densities.

The reliability of the PCEs was verified by conducting the stable power output measurements at the maximum power point,^47^ as shown in Figure S14. The improvement in the photocurrent is also confirmed by the external quantum efficiency (EQE) measurements (Figure 4b). The integrated current density of the CABI-10Br-based devices (4.67 mA cm^–2^) is higher than that of CABI (3.31 mA cm^–2^). The relative variation between the integrated and directly measured *J*_sc_ values is 5.4% for CABI ref and 3.3% for CABI-10Br-based devices, respectively. This could indicate that CABI ref devices exhibit relatively lower carrier collection at higher light intensities as compared to the CABI-10Br-based devices.^43^ Furthermore, an indirect verification of the results is possible from the dark current measurements (Figure 4c). The dark current for CABI-10Br-based solar cells is significantly lower than that of the CABI devices, indicating that the incorporation of Br^–^ at the I^–^ site indeed assists to suppress the defect/trap density and, hence, enhances the photovoltaic performance.^48,49^

We also correlated the EQE measurements with the reduction in trap-assisted recombination by measuring the light intensity-dependent *J–V* curves of the same solar cells.^43,50^Figure 4d displays the association between *V*_OC_ and light intensity (*P*) on a logarithmic scale.^43,44^ The primary process of charge recombination can be determined by the slope of the *V*_OC_*–*ln(*P*) curve.^51,52^ The Shockley*–*Read*–*Hall (SRH) recombination mechanism involves a charge carrier (electron or hole) recombining with a trap state in the material, releasing energy as heat rather than emitted light. The rate of SRH recombination depends on the density of trap states in the material as well as on the concentration of charge carriers. Bimolecular recombination is indicated by a slope near *kT*/*q*, whereas trap-induced charge recombination is shown by a slope near 2*kT*/*q*. The slopes were calculated to be 1.81 and 1.61 *kT*/*q*, for CABI and CABI-10Br devices, respectively, indicating that trap-induced charge recombination was hindered in the CABI-10Br-based devices. This further illustrates the relative reduction of DLD after Br^–^ incorporation.

**Figure 5:**
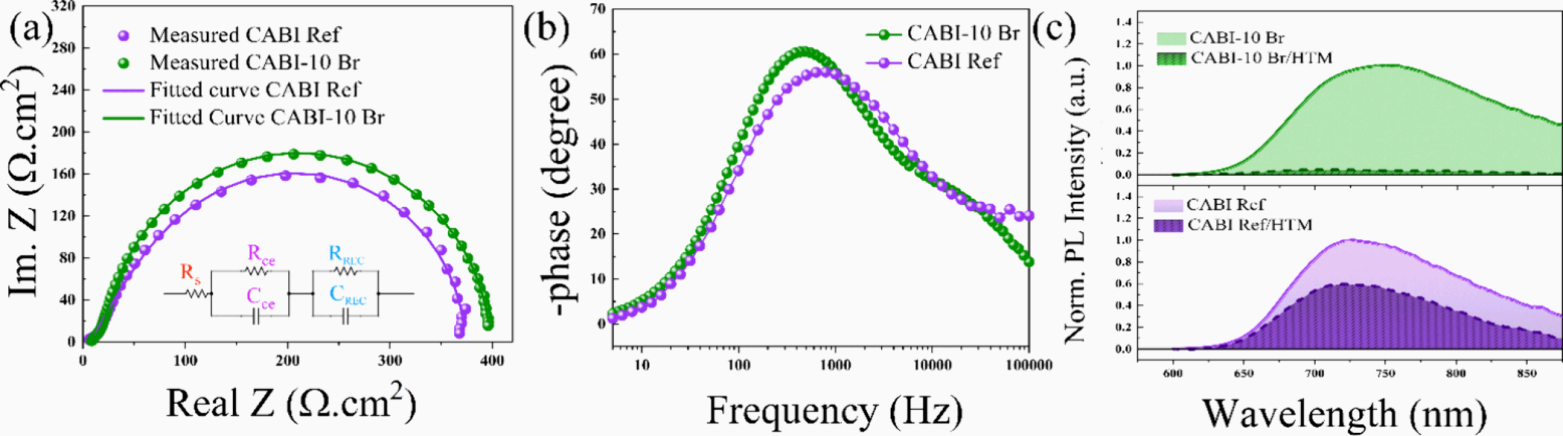
(a) Impedance spectroscopy Nyquist plots and (b) Bode phase angle plots for CABI ref and CABI-10Br devices and (c) PL spectra for CABI ref and CABI-10Br films with and without Spiro-OMeTAD HTM (hole-transport material), excited at 405 nm.

Impedance spectroscopy (IS) measurements were conducted to further probe the charge recombination in both cases. Figure 5a displays the results of the IS experiments with the fitting curves, whereas the inset highlights the equivalent circuit. *R*_s_ is the series resistance of the charge-collecting electrodes, whereas the two arcs that are observed are modeled with separate parallel RC elements. The high-frequency arc corresponds to the counter electrode charge transport resistance in the perovskite/HTM, while the low-frequency semicircle corresponds to charge recombination in parallel with chemical capacitance at the TiO_2_/perovskite interface.^53,54^ The counter electrode’s transport resistance and capacitance are denoted as *R*_ce_ and *C*_ce_, respectively, while the recombination resistance and capacitance are denoted as *R*_REC_ and *C*_REC_.^55,56^ CABI-10Br- and CABI-based devices exhibit similar *R*_s_ values of 8.53 and 8.77 Ω cm^2^, respectively, as expected for the same device structure. The *R*_ce_ values for CABI-10Br and CABI ref devices are 17.12 and 68.71 Ω cm^2^, respectively, while their corresponding *R*_REC_ values are 371.98 and 296.88 Ω cm^2^. The lower *R*_ce_ of CABI-10Br suggests an improved charge transfer at the counter electrode. Furthermore, the increase in R_REC_ indicates fewer recombination losses, thus leading to larger photocurrent. Based on the Bode phase angle plots shown in Figure 5b, the charge lifetime (τ_r_) in the perovskite film was also calculated according to the formula τ_r_ = 1/(2*f*_max_), where *f*_max_ denotes the frequency peak.^57,58^Table S7 summarizes the calculated values, demonstrating that the τ_r_ of the CABI-10Br-based device is longer than that of the reference CABI. The increased electron lifetime of the CABI-10Br suggests a lower charge recombination rate and higher charge collection efficiency. All the devices demonstrate similar geometric capacitances, indicating that the active layers’ dielectric constants were comparable despite having various phase compositions.^59^ Upon 10% Br mixing, the hole extraction at the CABI-*x*Br/HTM interface can be effectively promoted, as indicated by the larger photoluminescence quenching efficiency (*Q*% = 95.4%) compared with pristine CABI (*Q*% = 43.2%), as shown in Figure 5c. Similar analysis was done for CABI-5Br (*Q*% = 72.2%), illustrating a trend in PL quenching efficiency with addition of Br, as shown in Figure S15. We conclude that the enhancement in hole extraction in CABI-10Br significantly contributes to the increase in the *J*_sc_. Despite the recent efforts, the morphology of CABI films still appears not ideal for thin-film solar cells, which is a major reason for the poor performances when compared to, for example, Pb-based perovskite counterparts. The poor morphology is also an issue for other bismuth halide perovskite-inspired materials due to their rapid crystallization. However, the low toxicity of bismuth compositions, together with their superior film stability, makes them relevant for real-world applications once effective morphology engineering is carried out. Interesting approaches may include the addition of dopants, reducing agents, 2D structure components, and solvent engineering.^60^ In particular, we believe that “polymer-templated nucleation and growth (PTNG)” is a promising strategy to achieve desirable structures and orientations that lead to directional conductivity, as suggested elsewhere.^61,62^

## Conclusions

In summary, the incorporation of bromine (Br) in Cu_2_AgBiI_6_ (CABI) thin films can potentially reduce the trap density related to trap-assisted recombination, resulting in improved solar cells’ performance. The addition of bromine may possibly suppress the deep-level defects and, thus, lead to the formation of shallow transition energy levels. An improvement in photocurrent due to enhanced charge transfer dynamics is achieved by reducing the recombination losses. The reduced trap density results in an increase in the charge carrier lifetime, corresponding to the improvement of the external quantum efficiency. The fine-tuned morphology of CABI films upon Br incorporation also contributes to larger absorption coefficients and, hence, to the improved power conversion efficiencies of the corresponding solar cells. This work provides valuable insights into the halide engineering of Cu_2_AgBiI_6_. Though further investigation is required to fully understand the underlying mechanisms of trap mitigation and film crystallinity, this study presents a valuable approach for boosting the performance of Cu_2_AgBiI_6_-based solar cells. We are confident that, by leveraging the recent composition engineering strategies at the different sites of PIMs’ lattice, the potential of these emerging pnictogen-based absorbers for eco-friendly photovoltaics will be remarkably boosted.

## Experimental Procedures

### Materials

Bismuth iodide (BiI_3_), bismuth bromide (BiBr_3_), copper iodide (CuI), titanium diisopropoxide bis(acetylacetonate) (TDBA, 75 wt % in isopropanol), dimethylsulfoxide (DMSO), hydroiodic acid (HI, 57%), chlorobenzene (CB, extra dry, 99.8%), acetonitrile (99.9%), 4-*tert*-butylpyridine (4-tBP), and bis(trifluoromethane)sulfonimide lithium salt (LiTFSI, 99.95%) were purchased from Sigma-Aldrich. Tris[2-(1*H*-pyrazol-1-yl)-4-*tert*-butylpyridine]cobalt(III)tri[bis(trifluoromethane)sulfonimide] (FK209 Co(III), >98%) was purchased from Dyenamo. Dimethylformamide (DMF), silver iodide (AgI), and toluene were purchased from Alfa Aesar. 2,2′,7,7′-Tetrakis(*N*,*N*-di-*p*-methoxy phenylamino)-9,9-spirobifluorene (Spiro-OMeTAD) was purchased from Lumtec. Fluorine-doped tin(IV) oxide (FTO)-coated glass substrates of 2 cm × 2 cm were purchased from Yingkou Opv Tech New Energy Technology Co.

### Thin-Film Synthesis

We first prepared two solutions, namely, solution-A and solution-B as follows. Solution-A (for CABI ref) was prepared by mixing BiI_3_ (517.1 mg), CuI (238.1 mg), and AgI (241.8 mg) in 2 mL of DMSO:DMF (3:1) and heated at 150 °C for 45 min until it became clear and transparent. Solution-B was prepared by taking BiBr_3_ instead of BiI_3_, while all other compositions and procedures were maintained the same. CABI-*x*Br precursor solutions with varying Bi:Sb molar ratios were obtained by mixing solution-A and solution-B in the desired ratios. CABI ref and CABI-*x*Br films were deposited by spin-coating (at 3000 rpm for 1 min). The films were taken annealed in air for 50 min at 50 °C followed by increasing the annealing temperature to 150 °C. The samples were left to anneal for 4 min at 150 °C.

### Photovoltaic Device Fabrication

Pre-etched FTO substrates were sonicated for 15 min in each step with aqueous Mucasol solution (2% in water), deionized water, acetone, and 2-propanol followed by drying using nitrogen gas flow. On the as-prepared FTO substrates, a compact titanium dioxide layer (c-TiO_2_) layer (thickness, ∼50 nm) was deposited by spray pyrolysis at 450 °C of titanium diisopropoxide bis(acetylacetonate) solution (0.38 M) and then sintered at 450 °C for 1 h in air. The mesoporous TiO_2_ (mp-TiO_2_) layer was deposited by spin-coating (4000 rpm per 10 s) of 30NRD TiO_2_ nanoparticle paste/ethanol (0.3 g/1 mL) solution. The mp-TiO_2_-coated substrates were calcined at 450 °C for 30 min in air and then were immediately transferred into a N_2_-filled glovebox when they were at slightly above 150 °C. The CABI ref and CABI-*x*Br absorber layers were deposited on top of mp-TiO_2_ substrates. Spiro-OMeTAD (28 mM in CB) was doped with 14.5 μL of FK209 (300 mg/mL in acetonitrile), 8.75 μL of LiTFSI (520 mg/mL in acetonitrile), and 14.4 μL of 4-*tert*-butyl pyridine (tBP). The solution was deposited by dynamic spin-coating 80 μL at 1800 rpm for 30 s, after which the devices were stored overnight in a dry-air atmosphere. Finally, a 100 nm-thick gold electrode was thermally evaporated under vacuum (pressure, <10^–6^ mbar) via a metal shadow mask to obtain an active area of 20 mm^2^.

### XRD

XRD patterns were collected using a Malvern Panalytical Empyrean multipurpose diffractometer with a Cu Kα X-ray source (λ = 0.15418 nm). The X-ray tube was operated at 45 kV and 40 mA. The visualization system for the electronic and structural analysis (VESTA) program^58^ was used to draw the crystal structures. Structural information was derived from Rietveld refinement using the X'Pert Highscore software suite. The phase purity of the as-synthesized samples was estimated via Rietveld refinement of the XRD results with consideration of full refinement of the crystallographic and instrumental parameters.

### SEM

The SEM images of the films were collected using a field-emission scanning electron microscope (FE-SEM, Zeiss Ultra Plus, Carl Zeiss, Germany) operated at 3 kV. EDS spectroscopy (Oxford Instruments X-MaxN 80 EDS) in combination with a Zeiss Ultra Plus FE-SEM instrument was used to determine the elemental composition of the films.

### XPS

The XPS measurements were performed in ultrahigh vacuum employing an Al Kα X-ray source (*h*ν = 1486.7 eV) and an Argus electron spectrometer (Omicron Nanotechnology GmbH). For the measurement, perovskite film samples were spin-coated on FTO glass substrates. The chemical states of elements were determined from the XPS spectra by least-squares fitting of synthetic Gaussian–Lorentzian line shapes after background subtraction. The analysis was made in CasaXPS software, version 2.3.25PR1.0.59. Due to peak overlap, Cu 2p and Sb 3d were analyzed from Cu 2p_1/2_ and Sb 3d_3/2_ peaks instead of the more intense Cu 2p_3/2_ and Sb 3d_5/2_. The second doublet peak was fitted using a constrained peak area ratio (2:1 for p_3/2_:p_1/2_ and 3:2 for d_5/2_:d_3/2_) and peak separation (19.8 eV for Cu 2p and 9.34 eV for Sb 3d). The binding energy scale was calibrated according to C 1s (C–C/H) set to 284.8 eV.

### UV–Visible and PL Spectroscopy and Time-Correlated Single-Photon Counting (TCSPC)

UV–visible absorption spectra of the CABI and CABI-*x*Br films were collected on a Shimadzu UV-1800 absorption spectrometer (Shimadzu Corporation, Japan). An FLS1000 spectrofluorometer (Edinburgh Instruments, UK) was employed to measure steady-state PL spectra of the films. TCSPC measurements to obtain the TRPL decays were performed using a Picoharp 300 controller and a PDL 800-B driver for laser excitation. The excitation repetition rate was 200 kHz and controlled with a Tektronix function generator. A Hamamatsu R3809U-50 microchannel plate photomultiplier was used for detection in a 90° configuration.

#### Ultrafast TA Measurements

A Libra F laser system (Coherent, Inc.) produced 800 nm light pulses at a repetition rate of 1 kHz, which was split for the excitation and probe pulse generation in a roughly 90:10 ratio, respectively. The pulse width was ∼70 fs. The pump wavelength was tuned to 400 nm by a Topas C optical parametric amplifier (Light Conversion Ltd.) followed by decreasing the excitation energy density to 20 μJ cm^–2^ using neutral density filters. The white light continuum for the probe pulses was obtained by directing ∼10% of the primary 800 nm pulse energy to a water-filled cuvette. The measurement system (ExciPro, CDP, Inc.) comprised a silicon CCD, using an optical chopper for the pump pulses for reference measurements.

### Device Characterization

#### *J*–*V* and EQE Measurements

A Keithley 4250 source-monitor unit (4-wire sensing) was used to measure *J*–*V* curves under simulated solar radiation (100 mW/cm^2^ irradiance) illuminated by a class A++A+A LED powered solar simulator (SINUS-70 from Wavelabs). Calibration of the device to one sun intensity was performed using a Newport 91150-KG5 reference cell and meter. *J*–*V* curves were obtained under ambient conditions. A quantum efficiency measurement device (QuantX300, Newport) was used to collect the EQE spectra.

### IS Measurements

A potentiostat (Ivium Technologies B.V., CompactStat) was used to measure the IS of the devices. Impedance spectra were collected over the frequency range of 2 MHz to 1 Hz with an applied DC voltage of 0.5 V under dark conditions. The obtained IS data were fitted using EIS spectrum analyzer software based on the equivalent circuit model.

### Computational Details

DFT calculations with periodic boundary conditions (PBC) were performed using the basis set of numerical atom-centered orbitals (NAO),^63^ as implemented in the Fritz Haber Institute ab initio molecular simulations (FHI-aims) code.^64^ Within the FHI-aims framework, electrons were described by the zero-order regular approximation (atomic ZORA). A threshold of 1 × 10^–6^ eV was employed for self-consistency convergence of the total energy. The Perdew–Burke–Ernzerhof (PBE) exchange–correlation functional was employed for all geometry optimizations including the Tkatchenko–Scheffler (TS) correction^65^ accounting for van der Waals dispersion forces. Structures were relaxed until the maximum forces acting on each atom were below 0.02 eV Å^–1^. A 4 × 4 × 2 *k*-point sampling mesh was used for all models; these values ensure converged energies within 3 meV/f.u. The light-tier 1 basis of NAO was used for structural relaxations. The mixed occupancy of Bi/Ag/I/vacancy was simulated via the special quasi-random structure (SQS) approach as implemented in the Alloy Theoretic Automated Toolkit code.^66^ Electronic calculations were refined by means of the PBE0 hybrid functional to calculate the projected density of states (pDOS).

### Structural Models for DFT Calculations

The starting point for the computational model was the CABI structure (*R*3̅*m*, no. 166) provided by Sansom et al.^9^ We built an orthorhombic (2*a*, *a*+2*b*, *c*) supercell model, with Cu_2_AgBiI_6_ composition, containing 40 atoms (8 Cu, 4 Ag, 4 Bi, and 24 I). Random distribution and partial occupation of Cu, Bi, and Ag atoms and vacant sites were realized through a special quasi-random structure toolkit.^66^ Incorporation of Br in the structure (CABBrI) was built by the random replacement of I atoms with Br in the orthorhombic CABI supercell. The different Br contents, namely, 5 (CABI-5Br), 10 (CABI-10Br), 20 (CABI-20Br), and 40% (CABI-40Br), were obtained by randomly^66^ substituting I sites with 1, 2, 5, and 10 Br atoms, respectively. Theoretical models are depicted in Figure S1.

## Supporting Information Available

Structural models of the Br-doped orthorhombic CABI cell (4 Ag, 4 Bi, 8 Cu, and 24 I) with different bromine contents (1, 2, 5, and 10 Br atoms, respectively); lattice parameters of the rhombohedral CABI-Br unit cell with different Br contents; SEM images for CABI ref and CABI-10 Br films, thresholding using ImageJ; nominal expected compositions and calculated compositions from EDX spectra for the different precursor ratios; comparative EDS mapping for the different elements in CABI ref and CABI- 10Br samples; XPS survey spectra of CABI-10 Br and CABI ref XP high-resolution spectra of different elements; XPS analysis of surface composition for CABI ref and CABI-10Br samples; Tauc plot corresponding to direct allowed transitions and indirect allowed transitions for the different compositions; calculated band gaps for direct and indirect allowed transitions for the different compositions; band gap values calculated at the PBE0 level of theory; fitting results of TA decay data; computed impedance parameters; statistical distribution of PCE for different compositions; device statistics under standard 1 sun illumination; stable power output (SPO) of the devices near the MPP under continuous illumination; normalized PL spectra of CABI ref and CABI-5Br (PDF)
