# Supplementary material for: Halide Engineering in Mixed Halide Perovskite-Inspired Cu2AgBiI6 for Solar Cells with Enhanced Performance
Source: ACS Appl Mater Interfaces. 2024 Apr 3;16(15):19026–38. doi: 10.1021/acsami.4c02406 (PMC11040528; doi:10.1021/acsami.4c02406)
Supplement: Supplementary file 1 — am4c02406_si_001.pdf [file am4c02406_si_001.pdf]

Supporting Information (SI)

Halide Engineering in Mixed Halide Perovskite-Inspired Cu_2AgBiI_6 for Solar Cells with Enhanced Performance

Vipinraj Sugathan, ‡[1] *Maning Liu,* ‡#¶[1] *Adriana Pecoraro,*[2] *T. Kumar Das,*[3] *Tero-Petri Ruoko,*[4] *G. Krishnamurthy Grandhi,*[1] *Debjit Manna,*[1] *Harri Ali-Löytty,*[5] *Kimmo Lahtonen,*[6] *Ana Belén Muñoz-García,*[2] *Michele Pavone,*[7] *Paola Vivo*[1]*

AUTHOR ADDRESS

[1]Hybrid Solar Cells, Faculty of Engineering and Natural Sciences, P.O. Box 541, FI-33014 Tampere University, Finland

[2]Department of Physics "Ettore Pancini" University of Naples Federico II, Comp. Univ. Monte Sant'Angelo 80126 Naples, Italy

[3]Division of Materials Science and Engineering, Hanyang University, 222 Wangsimni-ro, Seongdong-gu, Seoul, 04763, Republic of Korea

[4]Smart Photonic Materials, Faculty of Engineering and Natural Sciences, Tampere University, P.O. Box 541, FI-33101 Tampere, Finland

[5]Surface Science Group, Photonics Laboratory, Tampere University, P.O. Box 692, FI-33014 Tampere University, Finland

[6]Faculty of Engineering and Natural Sciences, Tampere University, P.O. Box 692, FI-33014 Tampere, Finland

[7]Department of Chemical Sciences, University of Naples Federico II, Comp. Univ. Monte Sant'Angelo 80126, Naples, Italy

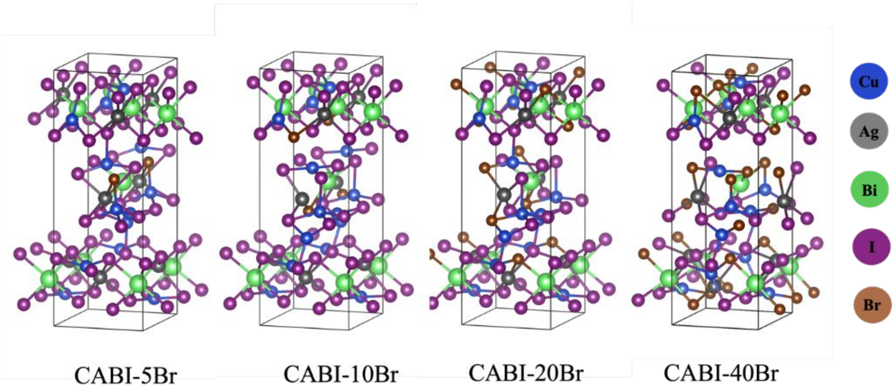

Figure S1. Structural models of Br-doped orthorhombic CABI cell (4Ag, 4Bi, 8Cu, 24I) with different bromine contents (1Br atom, 2Br atoms, 5Br atoms, 10 Br atoms, respectively). Structures have been optimized at PBE-TS level of theory. Color legend: Cu, blue; Ag, silver; Bi, green; I, violet, Br, brown.

Table S1. Lattice parameters of the rhombohedral CABI-Br unit cell with different Br contents. All structures are calculated at PBE-TS level of theory. Values in parentheses are experimental Rietveld refined values.

Lattice parameters (Å)	***CABI***	***CABI-5Br***	***CABI-10Br***	***CABI-15Br***	***CABI-20Br***	***CABI-40Br***
a=b	4.35 (4.33)	4.34	4.33 (4.29)	4.29	4.30	4.27
c	20.93 (21.19)	20.76	20.91 (20.95)	20.80	20.69	20.35

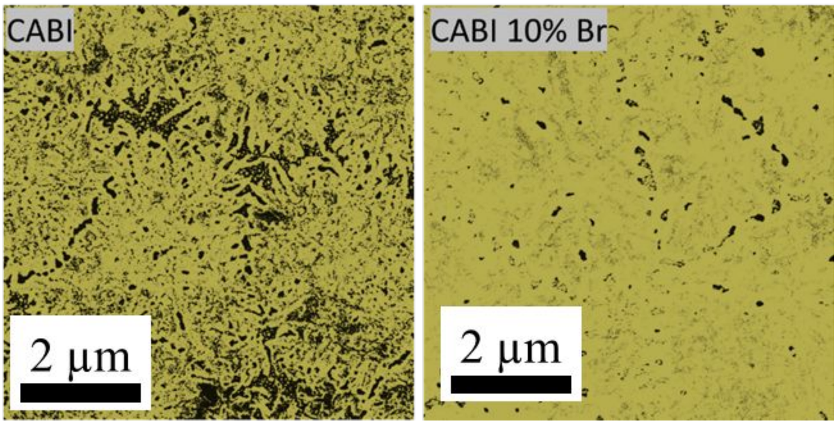

Figure S2. SEM images for CABI Ref and CABI-10 Br films, thresholding using ImageJ to understand coverage.

Table S2. Nominal expected compositions and calculated compositions from EDX spectra for the different precursor ratios.

	Nominal	***EDX (Calculated compositions)***	***%Br in Halide***
CABI Ref.	Cu_2AgBiI_6	$Cu_{2.12}AgBi_{0.76}Br_{0.04}I_{4.43}$	00.86 %
CABI-5 Br	$Cu_2AgBiBr_{0.3}I_{5.7}$	$Cu_{2.33}AgBi_{0.86}Br_{0.19}I_{5.19}$	03.51 %

CABI-10 Br	$Cu_2AgBiBr_{0.6}I_{5.4}$	$Cu_{2.26}AgBi_{0.79}Br_{0.41}I_{4.31}$	08.70 %
CABI-20 Br	$Cu_2AgBiBr_{1.2}I_{4.8}$	$Cu_{2.24}AgBi_{0.68}Br_{1.05}I_{3.69}$	22.08 %
CABI-40 Br	$Cu_2AgBiBr_{2.4}I_{3.6}$	$Cu_{3.47}AgBi_{0.32}Br_{1.36}I_{2.81}$	32.67 %

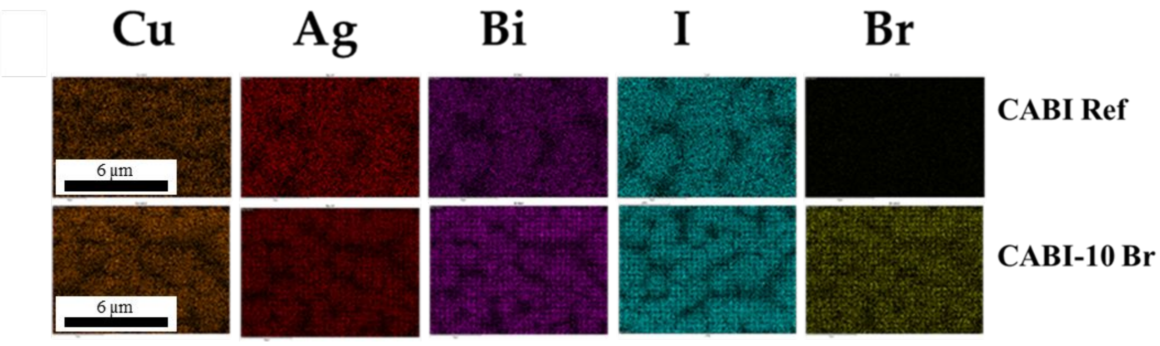

Figure S3. comparative EDS mapping for the different elements in CABI Ref and CABI- 10Br samples.

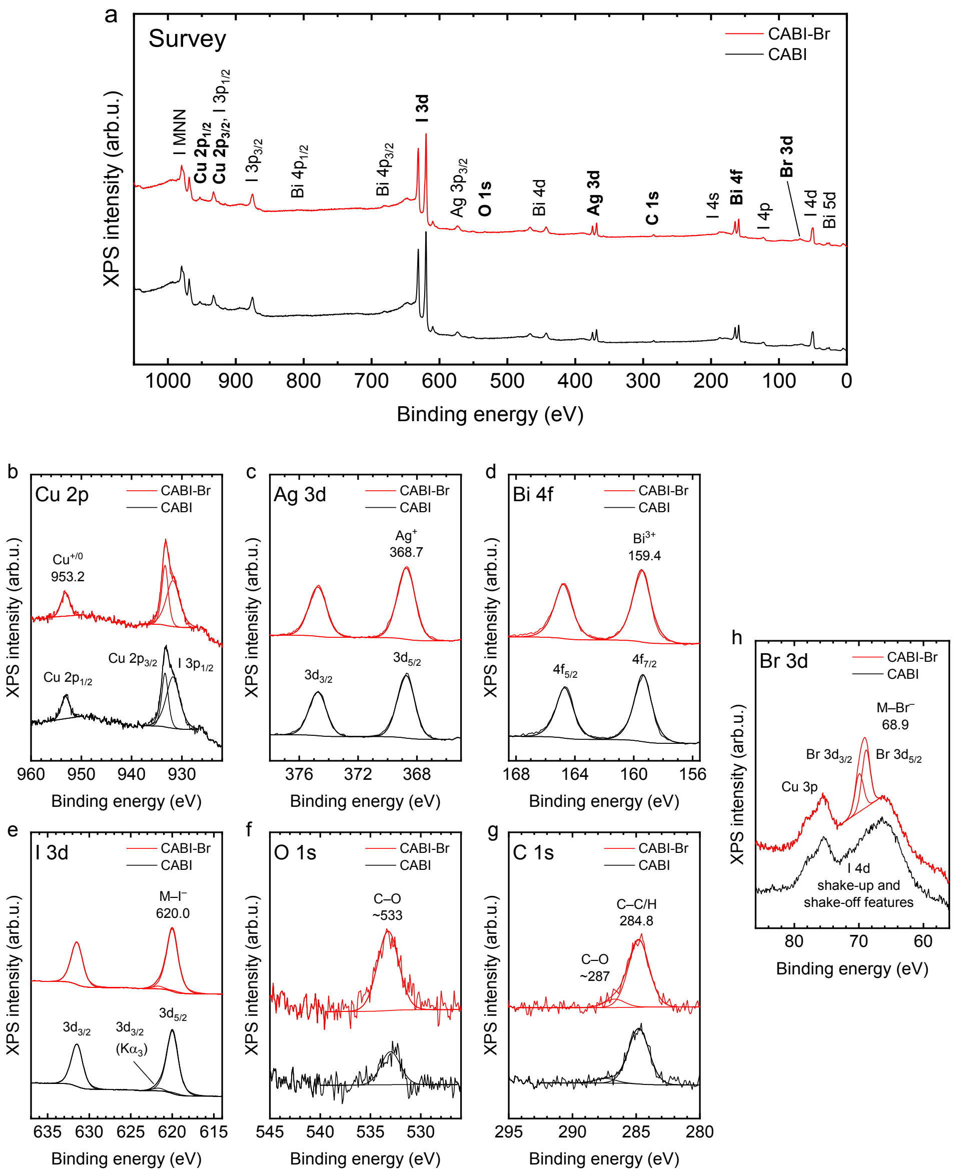

Figure S4. (a) XP survey spectra of CABI-10 Br and CABI Ref. XP high-resolution spectra of (b) Cu 2p, (c) Ag 3d, (d) Bi 4f, (e) I 3d, (f) O 1s, (g) C 1s, and (h) Br 3d for CABI-Br and CABI.

Table S3. XPS analysis of surface composition for CABI ref and CABI-10Br samples

At. %	$Cu^{+/0}$	Ag^{+}	Bi^{3+}	I (M-I⁻)	Br (M-Br⁻)	O(C-O)	C(C-C)	C(C-O)
CABI Ref	3.74	11.52	12.56	55.82	-	1.66	13.57	1.15
CABI-10Br	3.59	11.60	12.05	48.46	4.50	3.70	14.31	1.79

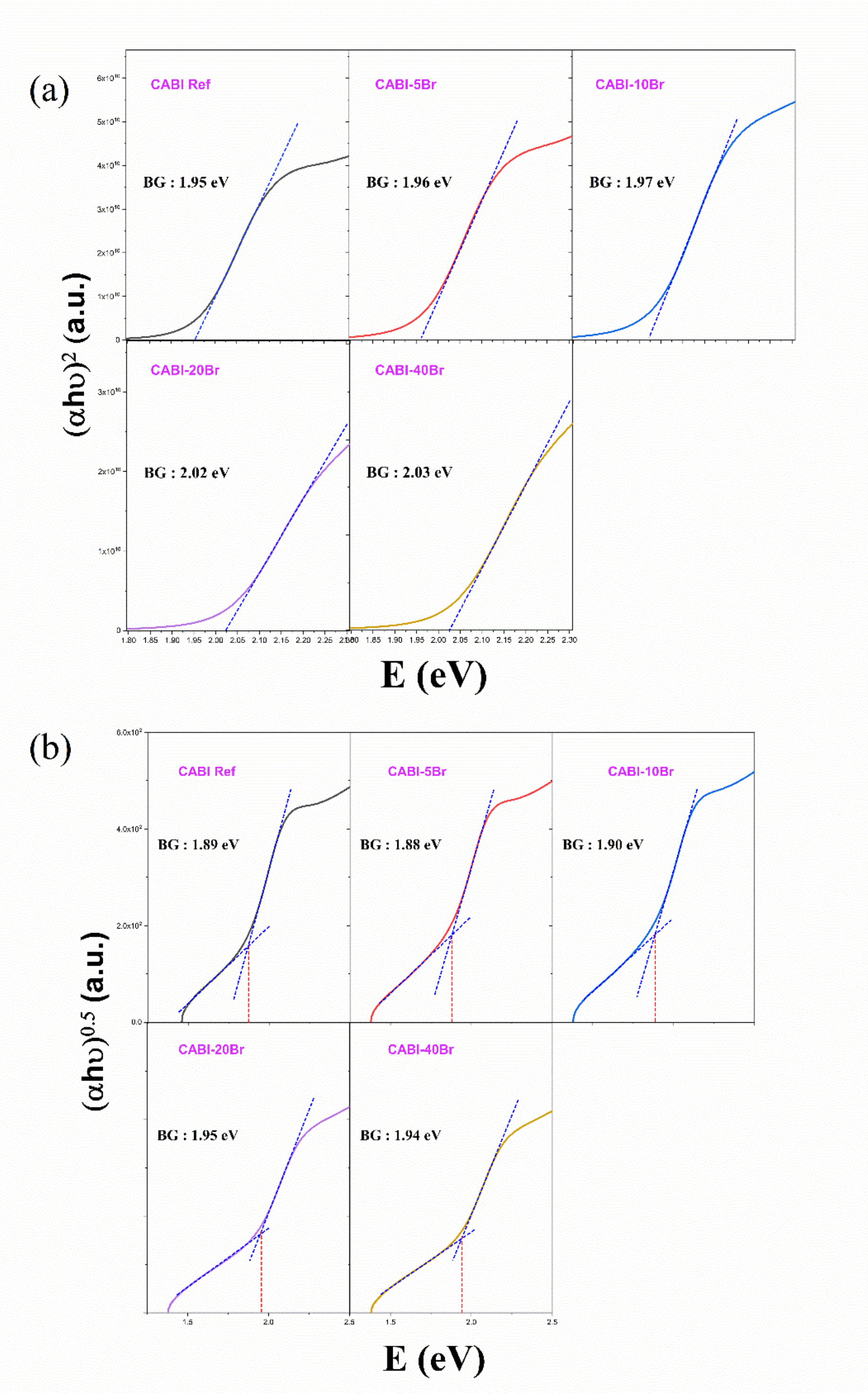

Figure S5. Tauc Plot corresponding to (a) Direct allowed transitions and (b) Indirect allowed transitions for the different compositions.

Table S4. Calculated bandgaps for direct and indirect allowed transitions for the different compositions.

Composition	Direct BG	Indirect BG	Difference of Indirect-Direct Band gaps
CABI ref.	1.95	1.89	0.06
CABI 5% Br	1.96	1.88	0.08
CABI 10% Br	1.97	1.90	0.07
CABI 20% Br	2.02	1.95	0.07
CABI 40% Br	2.03	1.94	0.09

Table S5. Bandgap values calculated at PBE0 level of theory. The theoretical predicted type (D/I) of transition is reported in parentheses.

Composition	Bandgap (eV)
CABI	1.62 (D)
CABI-5Br	1.64 (D)
CABI-10Br	1.66 (D)
CABI-20Br	1.59 (D)
CABI-40Br	1.56 (I)

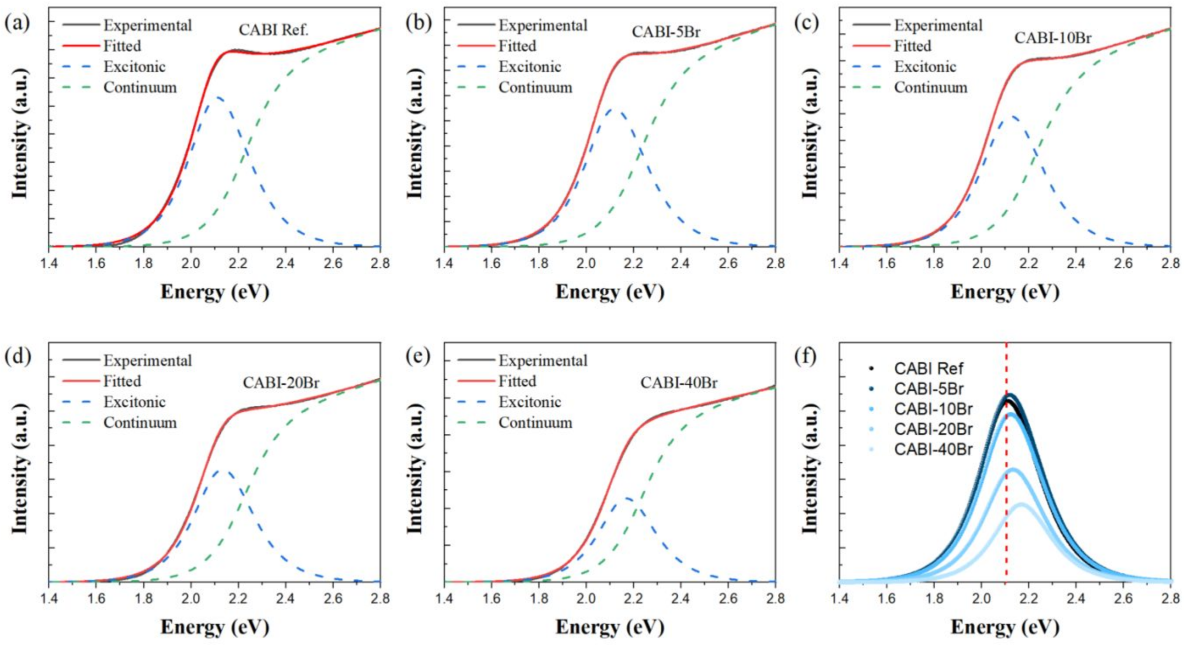

Figure S6. Elliott model fitting for absorbance of (a) pure CABI, (b) CABI-5Br, (c) CABI-10Br, (d) CABI-20Br, and (e) CABI-40Br thin films. (f) Comparative plot of fitted excitonic peak.

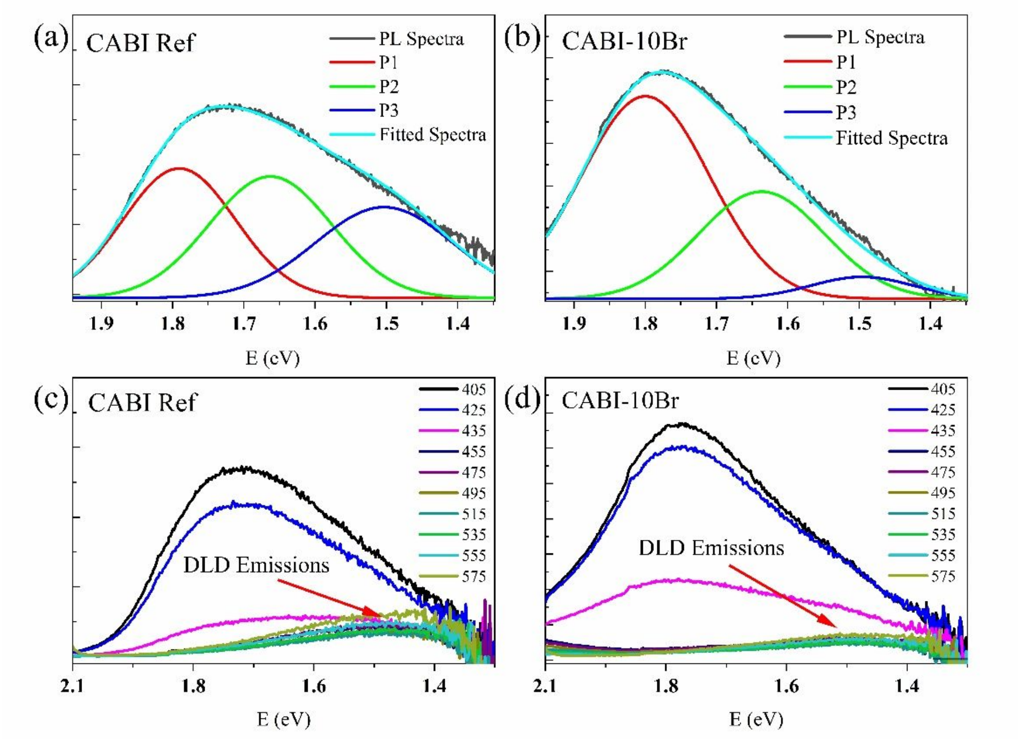

Figure S7. PL deconvolution for (a) pure CABI and (b) CABI-10Br, Excitation dependent PL for (c) pure CABI and (d) CABI-10Br.

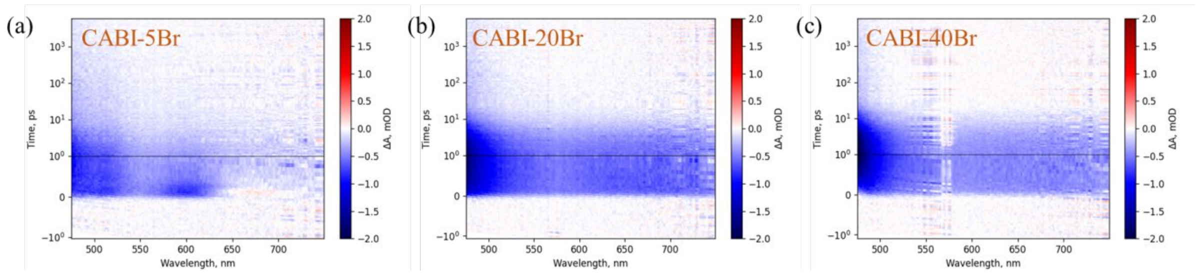

Figure S8. Transient absorption (TA) mapping images of (a) CABI-5Br, (b) CABI-20Br and (c) CABI-40Br films.

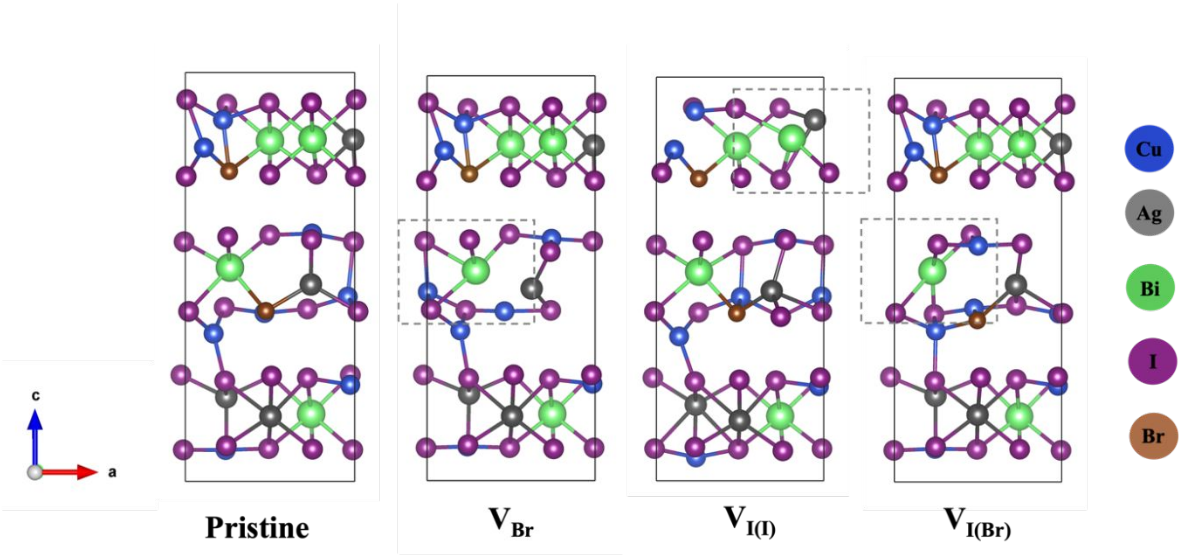

Figure S9. Optimized structures of different models of CABI-10Br; Pristine, Br-defective (V_{Br}) and two different cases of I-defective structures: I vacancy in a I octahedra $V_{I(I)}$ and in a mixed I/Br octahedra ($V_{I(Br)}$) .Octahedra where defect have been introduced are surrounded by a dashed rectangle.

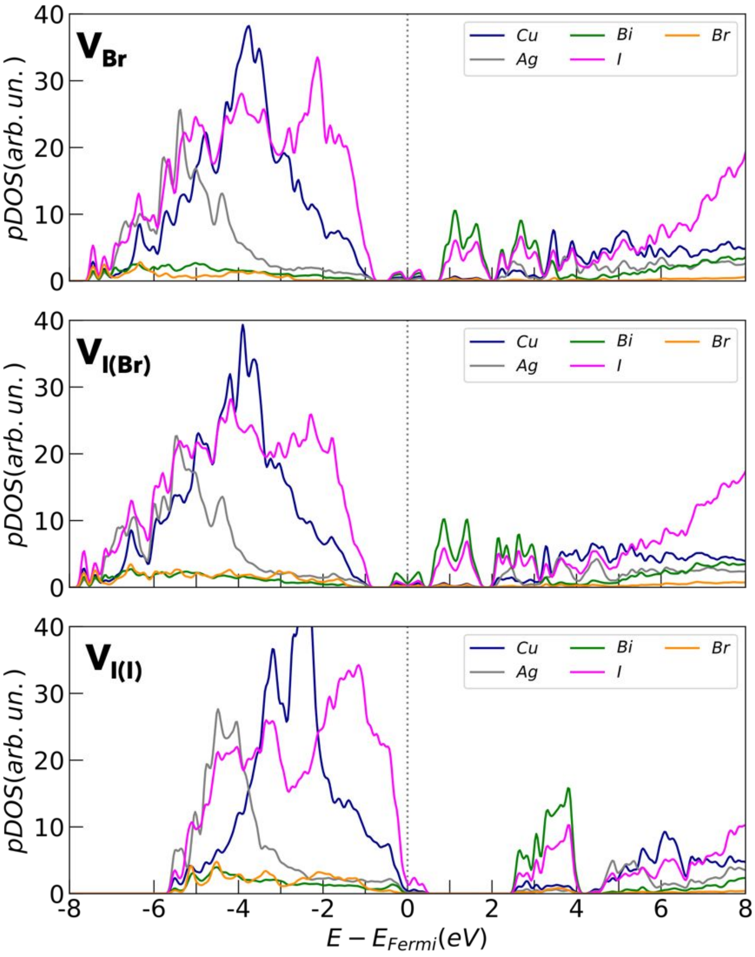

Figure S10. Atom-projected density of states (pDOS) calculated at PBE0 level of theory for the three different CABI-10Br halogen-defective models, V_{Br}, $V_{I(I)}$ and $V_{I(Br)}$.

Table S4. Fitting results of TA decays data.

	$\%A_1$	t_1 (ps)	$\%A_2$	t_2 (ps)	$\%A_3$	t_3 (ps)	$^{a)}t_{avg}$ (ps)
CABI Ref.	68.45	0.31	18.63	6.46	12.93	753.03	98.75
CABI-5Br	70.49	0.82	21.22	2.47	8.28	1556.33	130.04
CABI-10Br	7.31	0.52	88.92	5.46	3.77	3985.40	155.00
CABI-20Br	1.26	0.60	96.16	6.10	2.58	5285.61	142.13
CABI-40Br	7.30	0.65	91.50	6.98	1.20	6050.84	79.09

$^{a)}t_{avg} = \frac{A_1 t_1 + A_2 t_2 + A_3 t_3}{A_1 + A_2 + A_3}$

Table S5. Computed impedance parameters.

Parameters	*Ref*	*CABI-10Br*
R_s	8.77 Ω-cm^2	8.53 Ω-cm^2
R_{ce}	68.70 Ω-cm^2	17.12 Ω-cm^2
R_{REC}	296.88 Ω-cm^2	371.98 Ω-cm^2
C_{REC}	4.86 uF/cm^2	4.82 uF/cm^2
CPE^0	0.995	0.977
f_{max}	793.2 Hz	431.1 Hz
τ_r	0.201 ms	0.370 ms

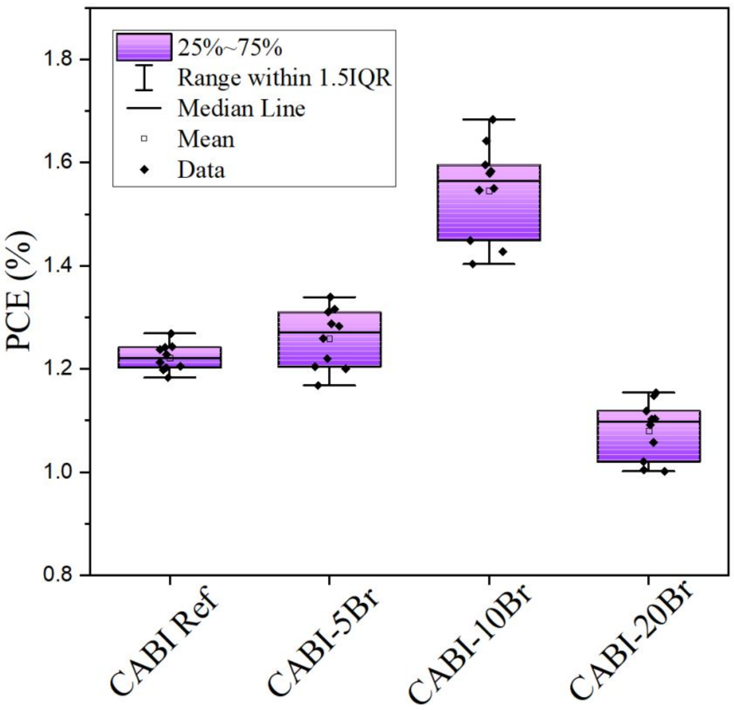

Figure S11. Statistical distribution of PCE for different compositions.

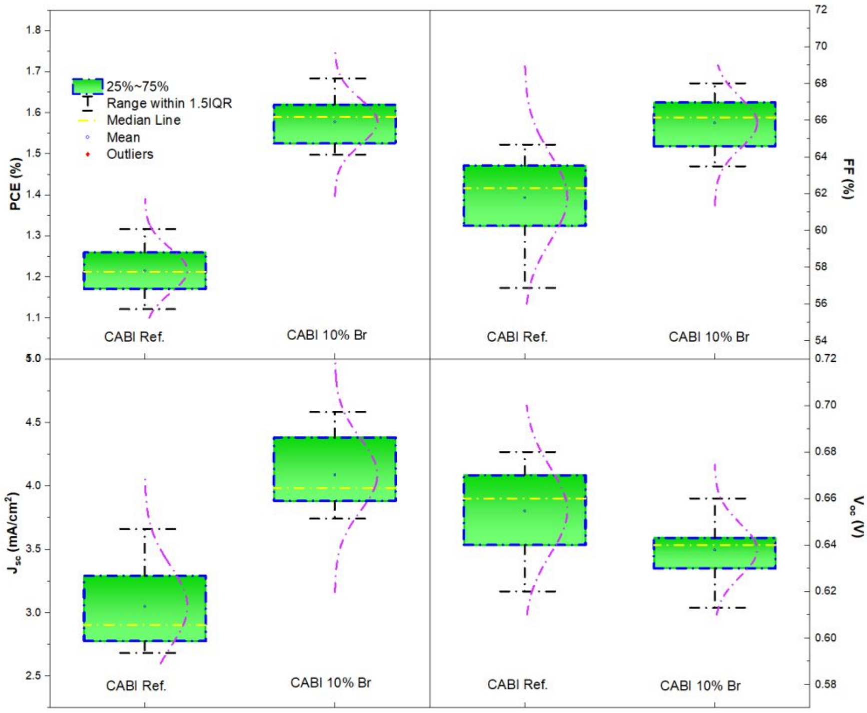

Figure S12. Device statistics Under standard 1 sun illumination.

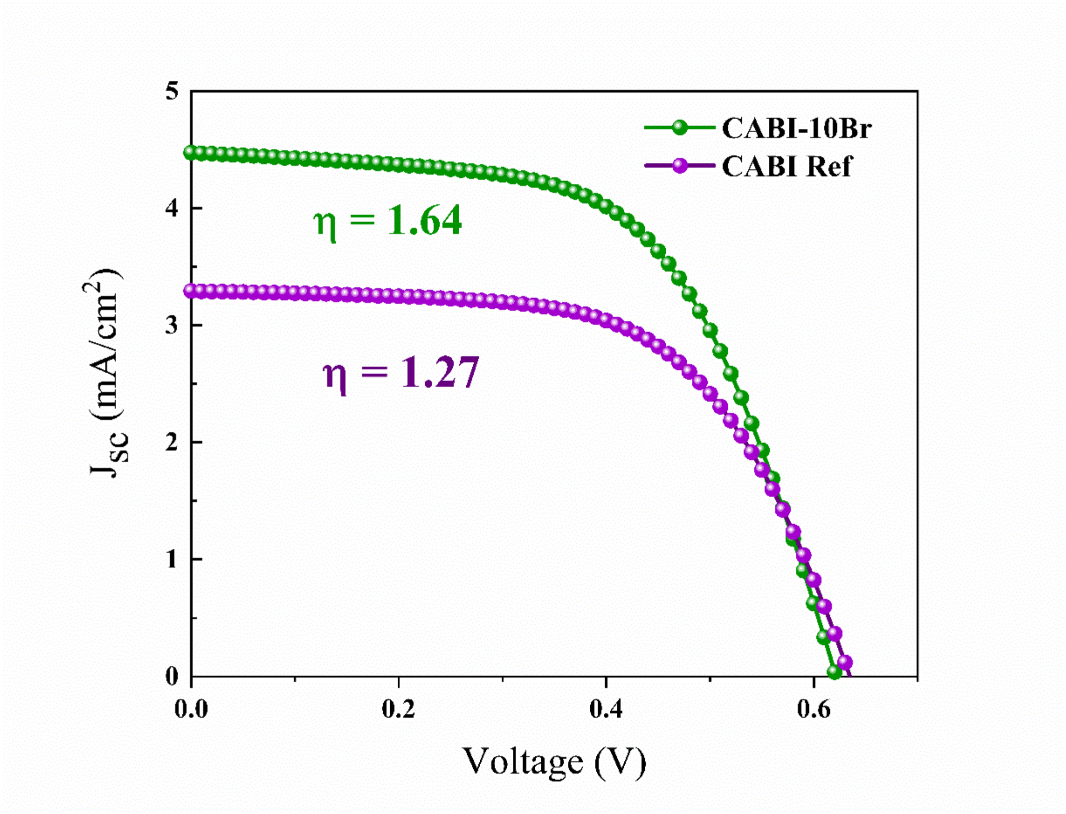

Figure S13. J-V plots under standard 1 Sun illumination, forward scan from 0 V to 0.7 V.

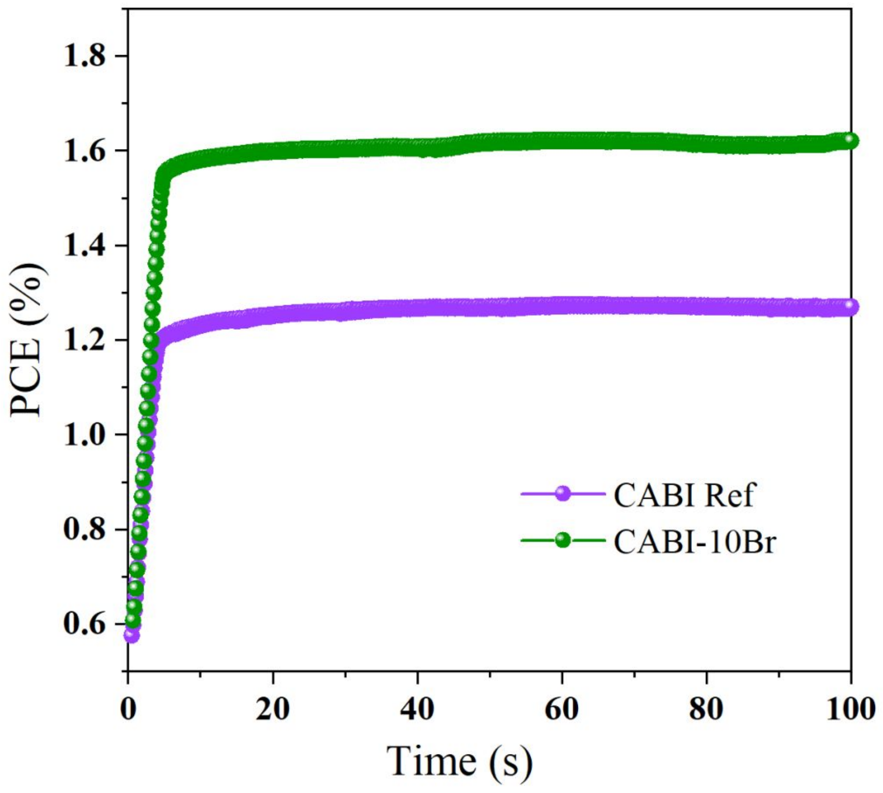

Figure S14. stable power output (SPO) of the devices near the MPP under continuous illumination (1 sun, 100mW/cm²).

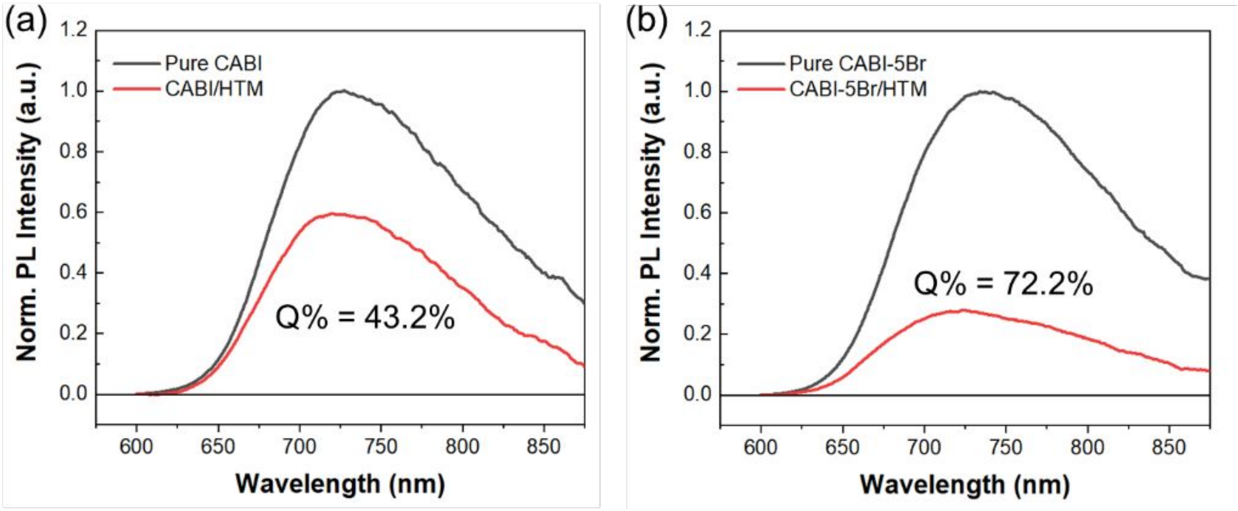

Figure S15. Normalized PL spectra of a) CABI Ref and b) CABI-5Br.
